# Innovative strategies for diabetic peripheral neuropathy: From clinical management to emerging bioengineering solutions

**DOI:** 10.1016/j.bioactmat.2026.02.023

**Published:** 2026-02-17

**Authors:** Zhi He, Jie Diao, Frederick G. Hamel, Bin Duan

**Affiliations:** a Mary and Dick Holland Regenerative Medicine Program, University of Nebraska Medical Center, Omaha, NE, 68198, USA; b Division of Cardiovascular Medicine, Department of Internal Medicine, University of Nebraska Medical Center, Omaha, NE, 68198, USA; c Department of Health Services Research & Administration, University of Nebraska Medical Center, Omaha, NE, 68198, USA; d Division of Diabetes, Endocrinology and Metabolism, Department of Internal Medicine, University of Nebraska Medical Center, Omaha, NE, 68198, USA; e Research Service, Nebraska-Western Iowa Health Care System, Omaha, NE, 68105, USA; f Department of Surgery, University of Nebraska Medical Center, Omaha, NE, 68198, USA; g Department of Mechanical Engineering, University of Nebraska-Lincoln, Lincoln, NE, 68588, USA

**Keywords:** Diabetic peripheral neuropathy, Drug therapies, Animal models, Biomaterial-based drug delivery systems, Microfluidic platforms, Bioengineered devices

## Abstract

Diabetic peripheral neuropathy (DPN) is a common, incurable complication of diabetes that causes sensory loss, pain, and motor problems. Conventional treatments like blood glucose management, pain relief, and neuroprotective drugs have limited success and do not prevent disease progression. Advances in neurobiology, regenerative medicine, and bioengineering have led to novel therapies that target underlying mechanisms and promote regeneration. Monitoring and evaluating the onset and progression of DPN are essential for effective clinical management. Given rapid advances in understanding DPN and developing new treatments, a comprehensive review that covers clinical progress, molecular pathology techniques, and emerging bioengineering strategies is both timely and essential. This review addresses: (1) DPN pathophysiology; (2) drug therapies from clinical trials since 2020; (3) animal models used in DPN research; (4) progress and challenges in biomaterial-based drug delivery systems; (5) developments and limitations of microfluidic platforms for DPN modeling; and (6) bioengineered devices used for DPN diagnosis and monitoring. Integrating clinical insights, molecular techniques, and bioengineering innovations seeks to create a forward-looking framework for next-generation DPN treatment and management.

## Highlights

• Overview of current understanding of DPN pathophysiology.
• Discussion of existing and emerging therapeutic strategies for DPN.
• Review of microfluidic models and their applications in DPN research.
• Exploration of bioengineered tools for DPN assessment and monitoring.
• Integration of clinical data, molecular analysis, and bioengineering advances.

## Graphical abstract

**Figure:**
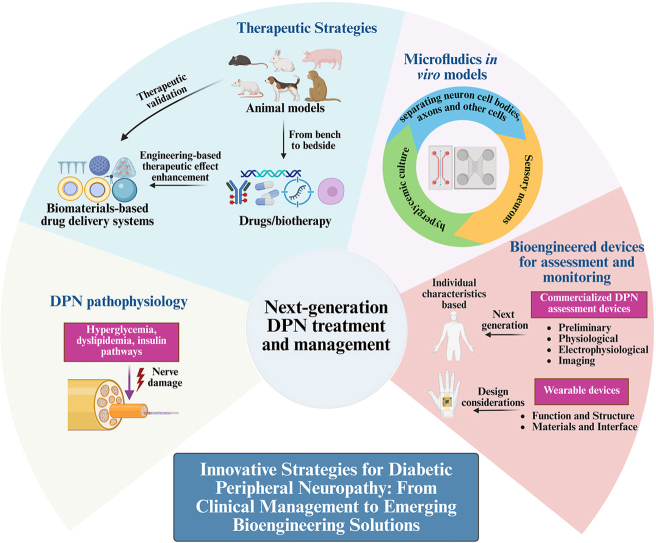

## 1 Introduction

Diabetic peripheral neuropathy (DPN), a prevalent chronic complication of diabetes, is associated with increased risk of sensory loss, neuropathic pain, foot ulcers, lower-limb amputation, depression, and anxiety, profoundly reducing quality of life [1]. The pathogenesis of DPN is complex, involving intricate interactions among hyperglycemia, obesity, oxidative stress, chronic inflammation, microvascular dysfunction, mitochondrial damage, and neurotrophic signaling disorders [2]. Current clinical treatments of DPN focus on symptom relief and pain management, with no approved effective therapies capable of preventing or reversing disease progression [3], underscoring the urgent need for mechanistic research and innovative therapeutic strategies.

Advances in neurobiology and regenerative medicine have expanded our understanding of DPN onset and regeneration mechanisms through both *in vitro* and *in vivo* molecular studies. Pharmacological advances have led to more translational clinical trials, while bioengineering innovations, especially biomaterials-based drug-delivery systems and microfluidics platforms, are fostering sophisticated therapeutic development, disease modeling, and mechanistic research. Numerous investigational drugs targeting pain pathways, metabolic regulation, inflammation control, and neurotrophic support are progressing in recent clinical trials, with biomaterial-based drug delivery systems offering unique advantages, like targeted delivery, sustained release, reduced systemic toxicity, and combination therapy [[3], [4], [5]]. Meanwhile, microfluidic platforms are emerging as advanced tools for DPN research, replicating the pathophysiological complexity of DPN by precisely controlling cell types, their origins, and the microenvironment within co-culture systems *in vitro*, thus facilitating mechanistic studies [6]. Assessing and monitoring DPN are equally vital for effective management. Bioengineered devices play a vital role in early DPN detection, enhanced risk stratification, and personalized treatment [7,8]. However, current review articles on DPN typically explore the disease from a single perspective or a limited number of perspectives. While providing in-depth insights in specific areas, they lack a unified framework. Given the complexity of DPN and the absence of a definitive cure, an interdisciplinary review is necessary.

This review elucidates the biological basis of DPN, evaluates current and emerging treatment options, and highlights the potential of biomaterial-based delivery systems, microfluidic technologies, and bioengineered devices designed for DPN assessment and monitoring. By integrating clinical advances, molecular pathology techniques, and innovative bioengineering approaches, we aim to bridge the gap between understanding disease mechanisms and applying these insights in clinical practice, thereby offering a forward-looking framework for next-generation DPN treatment and management.

## 2 Biological mechanisms of DPN

**Fig. 1:**
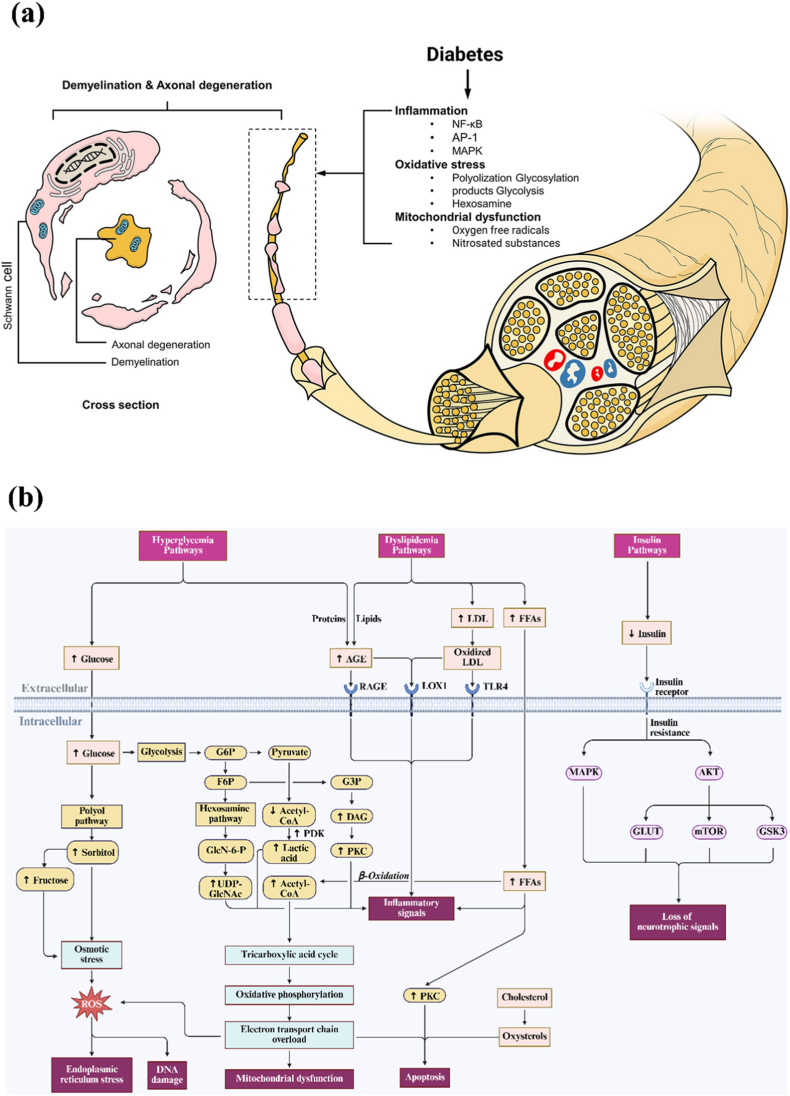
a) Diabetic peripheral neuropathy pathophysiology [14]; b) Major biological pathways of DPN, created by Biorender.

DPN occurs due to hyperglycemia, hyperlipidemia, and dysregulated insulin metabolism [9,10]. The pathogenesis of DPN involves metabolic dysfunction driven by diabetes-induced oxidative stress, inflammation, microvascular damage, and other factors, which disrupt nerve cell structure and function through specific signal transduction pathways, resulting in neuronal demyelination, neuronal damage, and ultimately peripheral neuropathy (Fig. 1a) [[11], [12], [13], [14]]. This stems from bioenergetic failure and mitochondrial dysfunction caused by excessive circulating glucose and lipids, providing a unified mechanism to explain the DPN's pathology and clinical manifestations [15]. Hyperglycemia and hyperlipidemia synergistically exacerbate DPN progression. Hyperglycemia alters cellular metabolic pathways, making peripheral nerve cells more sensitive to lipid toxicity, while abnormal lipid accumulation amplifies the damage caused by hyperglycemia [[16], [17], [18], [19]]. Additionally, although insulin does not affect glucose uptake in neurons, it can promote neuronal growth and survival. Dysregulated insulin metabolism caused by diabetes leads to a decrease in this neurotrophic signal, contributing to the development of DPN [20]. As shown in Fig. 1b, the biological mechanism of DPN can be generally summarized into three pathways: the hyperglycemia pathway, the hyperlipidemia pathway, and the insulin pathway.

### 2.1 Hyperglycemia pathways

Excess glucose is metabolized through one or more glucose metabolic pathways. Prolonged hyperglycemia disrupts these pathways, causing damage to nerve cells, vascular cells, and associated cells [20]. These pathways include the polyol pathway, the hexosamine biosynthetic pathway (HBP), the pyruvate dehydrogenase kinases (PDK) pathway, and the protein kinase C (PKC) pathway mediated by glycolysis, and the advanced glycation end products (AGEs) pathway [[20], [21], [22]].

#### 2.1.1 Polyol pathway

Under hyperglycemic conditions, glucose enters the polyol pathway after ingestion, where aldose reductase (AR) and sorbitol dehydrogenase (SDH) begin to function. Under the action of AR, glucose is converted into sorbitol using the nucleotide adenine dinucleotide phosphate (NADPH), and then nicotinamide adenine dinucleotide (NAD) is converted into nicotinamide adenine dinucleotide hydrogen (NADH) under the action of SDH [23]. Due to the slow diffusion rate of sorbitol in neural tissue and the lack of fructokinase that can metabolize fructose, sorbitol accumulates in both cells, resulting in an imbalance of osmotic pressure in the cytosol, osmotic stress, and subsequent excretion of myo-inositol and taurine. The lack of myo-inositol is directly related to the reduced activation of Na/K-ATPase and impaired cellular function. The reduction of taurine will lead to oxidative stress in Schwann cells [24]. Additionally, it also downregulates the glutathione reduction pathway, thereby inhibiting the clearance of free radicals and peroxides, ultimately forming ROS that aggravate nerve damage and NO-mediated vasodilation [25].

#### 2.1.2 Hexosamine Biosynthetic pathway (HBP)

The HBP originates from glycolysis, where glutamine-6-phosphate fructose aminotransferase (GFAT) catalyzes the conversion of fructose-6-phosphate to uridine diphosphate-N-acetylglucosamine (UDP-GlcNAc) [12,26]. Upregulation of GFAT during insulin resistance and/or diabetes leads to increased flux through HBP and increased synthesis of UDP-GlcNAc [27]. GlcNAc triggers oxidative stress in the endoplasmic reticulum (ER), while elevated fructose 6-phosphate (F6P) or glucosamine increases hydrogen peroxide levels and reduced the expression of insulin, glucose transporter 2 (Glut-2), and glucokinase genes [12,28]. Additionally, GlcNAc enhances transcription of the specific protein 1 (Sp1), which then activates transforming growth factor β (TGF-β) and plasminogen activator inhibitor 1 (PAI-1), contributing to microvascular complications [25]. Notably, only indirect evidence links the HBP to DPN pathogenesis, with no direct evidence available [26].

#### 2.1.3 Pyruvate Dehydrogenase Kinases (PDK) pathway

Glucose is metabolized to pyruvate via glycolysis, then transported into the mitochondria. Under the action of the pyruvate dehydrogenase complex (PDC), pyruvate is converted to acetyl-CoA for the TCA cycle. In diabetes, elevated acetyl-CoA/CoA and [NADH]/[NAD] ratios inhibit PDC activity and activate PDK. Following PDC inhibition, excess pyruvate is replaced by lactate [29]. Excess lactate promotes the pathogenesis of PDN by triggering reactive gliosis, macrophage infiltration, an acidic microenvironment, pro-inflammatory responses, and peripheral neuronal sensitization [25,30]. The PDK-PDH-lactate axis is a key component of the pathogenesis of inflammatory pain [31]. Restoration of pyruvate concentration promoted the recovery of DPN neurite damage [32].

#### 2.1.4 Protein Kinase C (PKC) pathway

Under hyperglycemia, the intermediate glyceraldehyde-3-phosphate (G3P) produced by glycolysis can be converted into diacylglycerol (DAG), thereby activating the neuronal PKC pathway. PKC is a serine or threonine kinase that binds to Ca^2+^-activated calmodulin and affects the function of other proteins [12]. PKC activation reduces Na/K ATPase in blood vessels and activates endothelial growth factor (VEGF), PAI-1, TGF-β, and nuclear factor kappa-B (NF-κB), leading to a series of microvascular complications such as inflammation, altered blood flow, basement membrane thickening, increased vascular permeability, and abnormal angiogenesis [25,33]. Downregulation of the PKC pathway has been shown to alleviate pain associated with DPN [34].

#### 2.1.5 Advanced Glycation End products (AGEs) pathway

When glucose and other sugars undergo non-enzymatic reactions, AGEs and their receptors (receptor for advanced glycation end products (RAGE)) are produced. Under high glucose conditions, AGE-RAGE levels increased and induced NF-kB-mediated inflammatory responses and apoptosis of Schwann cells [25]. At the same time, lipoprotein receptor-1 (LOX-1) acts as an AGE receptor and has higher LOX-1 ligand activity under diabetic conditions, leading to endothelial dysfunction and vascular lesions [35,36]. Reducing the production and accumulation of AGEs alleviated the pain caused by DPN [12].

### 2.2 Dyslipidemia pathways

In patients with type 2 diabetes mellitus (T2DM), hyperlipidemia is closely associated with DPN. Several potential mechanisms have been identified, including the low-density lipoprotein (LDLs) pathway, the free fatty acid (FFAs) pathway, and the cholesterol pathway. These pathways promote the production of inflammatory cytokines and induce neuronal apoptosis [20].

#### 2.2.1 Low-Density Lipoproteins (LDLs) pathway

LDLs can be converted into oxidized low-density lipoprotein (oxLDL) through oxidation and/or glycosylation. OxLDL can induce tissue damage by binding to three extracellular receptors, including endothelial cell type II membrane protein receptor LOX-1, Toll-like receptor 4 (TLR4, a pattern recognition receptor that initiates inflammatory and immune responses), and RAGE expressed in endothelial cells and Schwann cells, triggering a signaling cascade that activates NADPH oxidase and subsequent oxidative stress [20,36]. Additionally, Toll signaling has been shown to affect the development of early lesions in sensory neurons in DPN [36].

#### 2.2.2 Free Fatty Acids (FFAs) pathway

High plasma FFA levels are a hallmark of insulin resistance [37]. Palmitic acid is a common FFA that has been shown to directly cause Schwann cell damage and mitochondrial abnormalities in neurons *in vitro*. FFAs also have systemic effects, such as promoting the release of inflammatory cytokines from adipocytes and macrophages through β-oxidation [20,38]. Moreover, FFAs can upregulate PKC expression and induce endothelial cell apoptosis [37,39].

#### 2.2.3 Cholesterol pathway

Reduced serum cholesterol levels in patients with type 2 diabetes are associated with DPN [40]. Cholesterol may be oxidized to oxysterols, which are highly biologically active in regulating neuronal activation and have been shown to cause neuronal apoptosis and neuroinflammation [20,41].

### 2.3 Insulin pathways

Although neurons are relatively insensitive to insulin, insulin provides nutritional support to peripheral nerves and plays a direct role in the development of DPN [12,25]. Insulin receptors (INSRs) are expressed in neurons and Schwann cells within peripheral nerves. Insulin regulates pain, inflammation, and axon growth [42,43]. It activates phosphatidylinositol-3-kinase (PI3K) by inducing phosphorylation of insulin receptor substrate (IRS) proteins, which in turn activate mitogen-activated protein kinases (MAPKs) and protein kinase B (Akt) [42,43]. MAPKs include extracellular signal-regulated protein kinases (ERKs), p38 kinases, and c-Jun NH2-terminal kinases (JNKs), which activate damaged nerves through different molecular and cellular mechanisms, thereby affecting neuronal differentiation and apoptosis. Inhibition of the MAPK pathway can relieve pain [44,45]. Akt mediates glucose absorption by translocating glucose transporters (GLUTs) to the Schwann cells' plasma membrane. Downregulation of Akt under DPN conditions leads to ATP deficiency and reduced proliferation of Schwann cells [46,47]. Downregulation of Akt also reduces mTOR phosphorylation, leading to upregulation of DNA methyltransferase 1 (DNMT1) and a subsequent reduction in brain-derived neurotrophic factor (BDNF). Reduced mTOR-sterol regulatory element binding protein signaling also impairs myelin formation and induces sensory neuropathy [43,48]. Additionally, glycogen synthase kinase 3 (GSK3) is overactivated during periods of reduced insulin sensitivity, leading to inhibition of glycogen synthesis and further elevating blood glucose levels. This promotes endoplasmic reticulum stress-related apoptosis of Schwann cells and Tau phosphorylation, which slows the conduction velocity of motor neurons and disrupts the structure of myelin and axons [[49], [50], [51]]. The progression of DPN can be mitigated by regulating MAPK, GLUT, mTOR, and GSK3 pathways downstream of the insulin signal [50,[52], [53], [54]].

## 3 Therapeutic strategies for DPN

The development and research of therapeutic strategies for DPN bridges the gap between pre-clinical research and clinical application. This section covers current clinical treatments, preclinical *in vivo* animal models demonstrating therapeutic potential, and ongoing clinical trials of innovative drugs and biotherapy. Furthermore, we highlight the growing importance of biomaterial-based delivery systems in enhancing the precision, stability, and efficacy of drug delivery. Collectively, these efforts underscore the translation of novel therapies from pre-clinical research to the bedside, shaping future research directions.

### 3.1 Clinical treatments

The clinical treatment of DPN primarily aims to relieve or control the progression of pain in people with diabetes, encompassing both drug therapy and electrical stimulation therapy [25].

Clinical drugs used to treat pain caused by DPN mainly include antidepressants and anticonvulsants that inhibit abnormal nerve discharges. FDA (US Food and Drug Administration)-approved drugs for DPN include: (1) pregabalin, which limits Ca^2+^ influx during pain signal transmission; (2) duloxetine, which inhibits the transmission of neurotransmitter-mediated pain signals to the brain; (3) tapentadol, which binds to μ-opioid receptors in the spinal cord to reduce the perception of pain; (4) 8% capsaicin (only for foot analgesia), which binds to transient receptor potential vanilloid 1 (TRPV1) receptors to relieve pain [55,56]. Other clinically used drugs, including tricyclic antidepressants (TCAs), which relieve pain by antagonizing N-methyl-D-aspartate receptors that mediate hyperalgesia and allodynia; gabapentinoids, which reduce pain by inhibiting dorsal horn sensitivity through multiple mechanisms; venlafaxine, which inhibits sodium channels and increases norepinephrine levels in the central nervous system (CNS); sodium channel blockers, which block the activation of voltage-sensitive sodium channels; and low-dose naltrexone (LDN), which blocks Toll-like receptor 4 (TLR4) on microglia and thereby reduces neuroinflammation in the CNS [4,[56], [57], [58], [59]]. In addition to pharmacological treatments, nutritional supplements address metabolic dysregulation underlying DPN rather than merely relieving pain. These include alpha-lipoic acid, which reduces neurovascular abnormalities associated with DPN; benfotiamine, which inhibits the formation of AGEs; and vitamins, which prevent the worsening of DPN and diabetes-related symptoms [55,60].

In clinical practice, neuromodulation techniques offer alternative treatments for DPN by modulating nerve activity using electrical or magnetic energy. Currently, FDA-approved devices for managing DPN include transcutaneous electrical nerve stimulation (TENS), percutaneous electrical neurostimulation (PENS), magnetic peripheral nerve stimulation (mPNS), and spinal cord stimulation (SCS) [57,[61], [62], [63]]. TENS non-invasively stimulates A-β fibers through a wearable electrical stimulation device (SENSUS®, NeuroMetrix) to inhibit nociceptive transmission in the spinal cord [64]. For PENS, the First Relief (DyAnsys) auricular nerve stimulator relieves pain by applying periodic low-intensity electrical pulses through the skin to the peripheral nerve branches in the ear region [4,55]. mPNS operates by emitting low-frequency pulses that stimulate action potentials and neuronal activity in the ascending and descending pathways of the PNS and CNS, thereby reducing pain. Neuralace Medical's Axon Therapy (NeuraLace Medical) is the first FDA-approved non-invasive magnetic peripheral nerve stimulation treatment for painful DPN [62,65]. Compared to TENS, PENS, and mPNS, SCS is more commonly used for pain relief in DPN. Low-frequency SCS (10-100 Hz) induces paresthesia in overlapping pain regions to relieve pain by activating large fibers, whereas high-frequency SCS (1-10 kHz) stimulates inhibitory neurons in the dorsal horn, thereby modulating pain pathways [4,66]. Currently, several high-frequency SCS devices are approved by the FDA, including Nevro HFX, Intellis, Vanta (Medtronic), Senza II (Nevro), and Proclaim™ XR (Abbott), with additional systems anticipated in the near future [57]. Apart from these stimulation methods, no FDA-approved devices are available for treating DPN with transcranial direct current stimulation (tDCS) or repetitive transcranial magnetic stimulation (rTMS). Nonetheless, tDCS (Flow Neuroscience) and rTMS (Neurostar) are approved for the treatment of depression, and rTMS is being investigated in clinical trials for the treatment of DPN [67,68]. Acoustic energy may also serve as a neuromodulator, but only clinical trials using low-intensity focused ultrasound are ongoing, with evidence of effectiveness in alleviating neuropathic pain [69].

It is worth noting that no current drugs specifically target the pathogenesis of DPN; existing treatments only alleviate pain [3]. Given that diabetes is a systemic metabolic disease, future therapeutic strategies should aim to prevent neuropathic pain, reverse nerve damage, and control diabetes progression. Moreover, polypharmacy concerns (average 5.9 medications/diabetes patient in the USA) necessitate attention to adverse drug reactions (ADR) and individual variability [57].

### 3.2 Investigational translational and preclinical therapeutic development

**Fig. 2:**
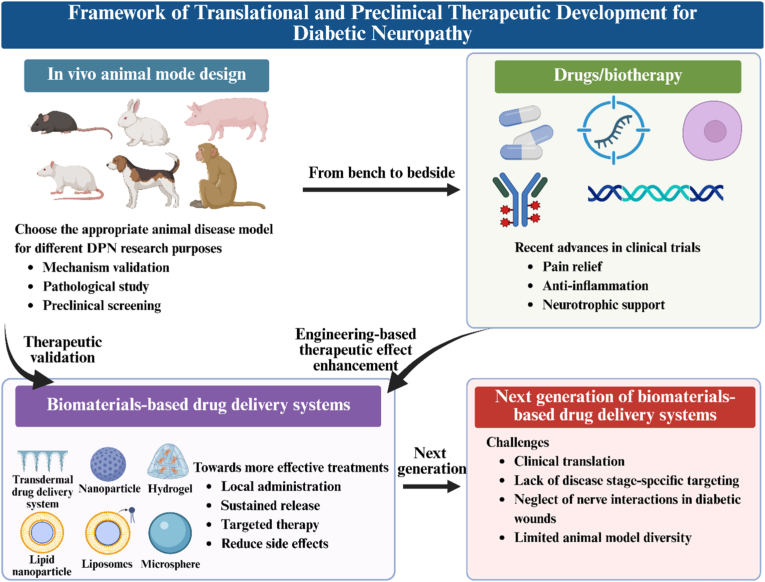
Framework of translational and preclinical therapeutic development for diabetic neuropathy, created by Biorender.

Translational and preclinical therapeutic development are driving innovation in the development and application of new therapeutic strategies. First, animal models provide crucial insights into disease mechanisms and efficacy. Next, emerging drugs and biological therapies, studied in clinical trials over the past five years, highlight the direction of recent clinical research. Finally, biomaterial-based drug delivery systems are summarized, focusing on achieving precise targeting, sustained drug release, protection, and reduced side effects. These advancements underscore the progression of translational and preclinical therapeutic development toward precision medicine, interdisciplinary integration, and personalized intervention. We hope to provide insights into the design of next-generation biomaterial-based drug delivery systems from the perspective of disease research in animal models and clinical studies (Fig. 2).

Selecting an appropriate DPN animal model facilitates validation of disease mechanisms and treatments, which is crucial for preclinical DPN research. Current biomaterial-based drug delivery systems can deliver appropriate drugs for localized sustained release, targeted therapy, and reduced side effects. The development of next-generation biomaterial drug delivery systems must consider their potential for clinical translation, stage-specific targeting of DPN, metabolic complexity, and animal model diversity to advance engineering-based DPN therapeutic innovations.

#### 3.2.1 *In vivo* animal models

Preclinical studies in animal models are crucial for understanding DPN mechanisms and developing new therapies. Nutritional induction, chemical induction, and genetic models are the three main strategies for establishing DPN in animal models [70]. DPN progression in animal models includes early (metabolic/neurological function changes, such as hyperalgesia), mid (structural damage, such as slowed nerve conduction velocity and intraepidermal nerve fiber loss onset), and late stages (irreversible neurodegeneration, such as axonal atrophy and demyelination) [71]. Rodent models are widely used to study DPN. The details of the types of rodent models and diabetic models, the study window time, the induction mechanism of DPN, and the advantages and disadvantages are summarized in Table 1.

**Table 1:** Overview of commonly used rodent models of DPN.

Types of rodent models	Diabetic types	Study time window	Induction mechanism of DPN	Advantages	Disadvantages	Ref
Nutrition-induced mouse	Type 2 diabetes	Early stage	Diabetic neuropathy caused by a high-sugar, high-fat diet	Symptoms of neuropathy, such as abnormal nerve conduction, heat sensation, and pain sensation at the early stage	Not applicable to the study of mid- to late-stage DPN	[70,72]
STZ-induced rodent	High dose: Type 1 diabetes; moderate dose: Type 2 diabetes	Early stage, moderate stage, late stage	Neuropathy due to abnormal glucose transport and glucokinase function, and beta cell destruction leading to insulin deficiency and hyperglycemia	Demonstrates symptoms of DPN from early to late stages; widely used in a variety of animal species; low cost and easy to optimize	STZ has certain neurotoxicity; a high mortality rate	[70,82]
ALX-induced rodent	Type 1 diabetes	Early stage	Rapid induction of hyperglycemia leading to neuropathy by damaging pancreatic β cells through the generation of reactive oxygen species	Significant symptoms of DPN at the early stage; low cost, and easy to optimize	The reactive oxygen species produced by ALX have certain neurotoxicity and can only cover the early DPN	[74,75]
AKITA mouse	Type 1 diabetes	Early stage, moderate stage, late stage	Mutation of the Ins2 gene leads to hyperglycemia after stress-induced apoptosis of β cells: Decreased sensory nerve conduction velocity, sympathetic hyperactivity, and impaired nociception	widely accepted T1DM-DPN model; clear inheritance; diabetes can last up to 8 months	Non-immune mechanism; females are phenotypically weaker than males; hyperglycemia only	[[84], [85], [86]]
ZDF rat	Type 2 diabetes	Early stage, moderate stage, late stage	Mutations in the leptin receptor lead to elevated circulating leptin levels, insulin resistance, and subsequent neuropathy	With hypertension and significant myelin damage	High mortality rate after 4-6 months; Failure to replicate neuroaxonal dystrophy (NAD)	[[100], [101], [102]]
SHR rat	Type 2 diabetes	Early stage, moderate stage, late stage	Neuropathy caused by hypertension	Mimics metabolic syndrome; prominent vascular lesions for the vascular-neural interactions study	No myelinated fiber loss or changes in fiber density	[103]
db/db mouse	Type 2 diabetes	Early stage, moderate stage, late stage	Leptin receptor mutations lead to obesity and thus DPN	widely accepted T2DM-DPN model; high degree of DPN simulation; prominent vascular lesions for vascular-neural	Death within 24-30 weeks of diabetes onset without insulin supplementation; background dependence (more severe in BKS than C57); Spontaneous remission of sugar level in the later period	[70,101,104]
ob/ob mouse		Early stage, moderate stage	Lack of functional leptin leads to obesity and thus DPN	Hyperinsulinemia; marked decrease in IENF density and motor and sensory nerve conduction velocities	Death within 24-30 weeks of diabetes onset without insulin supplementation; mild hyperglycemia ; atypical DPN phenotype	
MKR mouse	Type 2 diabetes	Early stage, moderate stage	Muscle-specific IGF-1R mutations lead to systemic insulin resistance and DPN	Directly simulates muscle insulin resistance; no obesity interference; sensory and motor function impairment	Relatively mild neuropathy; requires behavioral testing	[105,106]
NOD mouse	Type 1 diabetes	Early stage, moderate stage	DPN results from spontaneous autoimmune destruction of β cells	Genetics and immunology are similar to human diseases; prominent insulitis; nonobese; severe neuropathy develops faster and more severely than in other rodent models	Large individual differences; limited progression of DPN (primarily changes in thermal allodynia); pathogen-free conditions required; other autoimmune diseases (such as thyroiditis)	[72,82,107,108]
BB rat	Type 1 diabetes	Early stage, moderate stage, late stage	Autoimmune β-cell damage inducing DPN	Severe insulin deficiency; highly similar to human T1DM	Expensive; insulin supplementation is required as the symptoms worsen; Signs of lymphocytopenia; other autoimmune diseases (such as glomerulonephritis)	[82,101,109]
OLETL rat	Type 2 diabetes	Early stage, moderate stage	Cholecystokinin A receptor (CCK-A) deficiency leads to obesity, insulin resistance, and subsequent DPN	Similar to human non-insulin-dependent diabetes mellitus (NIDDM); significant autonomic neuropathy	DPN onset late; only in males	[74,110,111]

Nutritional induction from a high-fat or high-sugar diet leads to the development of DPN in type 2 diabetes. C57BL/6 mice exhibit neuropathic symptoms, including abnormal nerve conduction and altered thermal and pain sensation, but are primarily used for prediabetes and obesity-related neuropathy rather than chronic DPN due to the absence of intradermal nerve fiber loss or axonal atrophy [70,72]. Therefore, nutritional induction alone is not a standard model for DPN. In contrast, chemical induction and genetic modification are more frequently used in DPN animal models.

Chemical induction of DPN is usually achieved through streptozotocin (STZ) or Alloxan (ALX). STZ interferes with glucose transport and glucokinase function, selectively destroying β cells in the pancreas. A single high-dose injection of STZ causes insulin deficiency and hyperglycemia, mimicking type 1 diabetes, while a moderate-dose injection partially damages pancreatic β cells, mimicking type 2 diabetes [70,73,74]. ALX damages pancreatic β cells by generating ROS, thereby inducing hyperglycemia. Early DPN symptoms, including hyperalgesia and allodynia, appear in both STZ and ALX diabetic rodent models. STZ also exhibits DPN late-stage features, including hypoalgesia, ataxia, neurodegeneration, epidermal nerve fiber loss, and demyelination [74,75]. Additionally, nicotinamide (NA) can be used to partially protect pancreatic β cells from the effects of STZ and prevent the development of non-obese type 2 diabetes in mice [70]. STZ-induced DPN has been established in multiple species, including mice, rats, pigs, monkeys, and others. [73,76]. Large animal models better simulate human DPN. In the STZ pig model, gastrointestinal nervous system disorders, a significant decrease in the number of small-diameter myelin fibers, increased RAGE and S100B immunoreactivity, increased myosin (MG) levels, and heart rate changes have been reported [[77], [78], [79]]. In the STZ monkey model, decreased motor nerve conduction velocity (MNCV), prolonged F wave latency, and nerve fiber atrophy occurred [80]. However, no microvessel changes were shown in the *Macaca fuscata* model [81]. Compared with other diabetes modeling approaches, chemical induction is cost-effective and easy to optimize [70], although STZ has certain neurotoxicity and high mortality [76,82].

Genetic models of DPN are mainly rodent based (Table 1) [80]. Genetically engineered models include AKITA mice, Zucker diabetic-fatty (ZDF) rats, obese spontaneously hypertensive (SHR) rats, diabetic mice (db/db) and obese mice (ob/ob), and muscle IGF-I receptor-lysine-arginine (MKR) mice, while spontaneous models include non-obese diabetic (NOD) mice, biologically bred (BB) rats prone to diabetes, and Otsuka Long-Evans Tokushima Fatty (OLETL) rats. Among them, AKITA and db/db mice are most used [70,75,83]. AKITA mice, which develop insulin misfolding due to an Ins gene mutation, are widely used for type 1 diabetes research. The duration of diabetes can reach 8 months, far exceeding the 5-8 weeks maintained in NOD [84]. AKITA mice have decreased sensory nerve conduction velocity, sympathetic nerve hyperexcitability, and impaired nociception [85,86]. db/db mice, a common type 2 diabetes model due to the mutation of the leptin receptor, with obvious hyperglycemia and developing DPN at 8 weeks of age, showing prolonged thermal latency, slowed nerve conduction velocity, and loss of intraepidermal nerve fiber (IENF) density [87]. Additionally, smaller transgenic animal models such as zebrafish are widely used to study the DPN mechanism [82,88]. Although no transgenic DPN pig or monkey models currently exist, several transgenic diabetic pig models have been established, including dominant negative glucose-dependent insulinotropic polypeptide receptor (GIPRdn), hepatocyte nuclear factor-1α (HNF1A), INSC94Y, INSC93S transgenic pig, and others. [89,90]. Notably, INSC94Y transgenic pigs showed no neurological lesions during a one-year observation period [91]. The future application of CRISPR/Cas9 could improve the precision and efficiency of developing new models [82,92]. Moreover, humanized rodent models minimize species gaps for preclinical evaluation [82]. Compared to other approaches, genetic models provide greater disease specificity, reproducibility, and controllable genetic backgrounds, making them more ideal for complex pathological studies. However, the limitations include high cost, lengthy development timelines, unstable phenotype, and potential off-target mutations [93].

In general, animal models of DPN have successfully reproduced key pathophysiological characteristics, provided controllable experimental conditions, facilitated drug screening platforms, and made tissues accessible [83]. However, no single model captures all clinical features of DPN. Researchers should select appropriate animal models to evaluate the therapeutic effects or potential mechanisms of DPN, depending on the different research objectives, while acknowledging that species and experimental differences limit the clinical relevance [80,83]. Additionally, not only are non-human primate models rapidly being phased out due to political pressure and expense, but the FDA is gradually reducing animal testing in preclinical drug safety studies by using *in vitro* platforms or computational modeling [94,95]. *In vitro* models can address the limitations to a certain extent: 1) 2D co-culture systems of neurons, endothelial cells, immune cells, pancreatic islet cells, and 3D organoid co-culture systems help dissect intercellular communication in nerve injury; 2) Patient-derived induced pluripotent stem cell (iPSC) models simulate DPN in a genetic background; 3) Microfluidic chips create a more biomimetic microenvironment with 3D neural cell bodies and axons in controllable culture conditions [[96], [97], [98], [99]]. Nevertheless, given the systemic nature of diabetes and the limited ability of *in vitro* models to simulate systemic pathological changes and behavioral alterations, they cannot wholly replace animal models in DPN research [83]. Future research on DPN will integrate *in vivo* and *in vitro* models to achieve mechanistic insights and translational potential.

#### 3.2.2 Drugs and biotherapy in clinical trials

Existing treatments mainly offer pain relief but have a limited impact on reversing DPN progression. This highlights the urgent need for mechanism-based therapies. We summarize recent clinical studies since 2020 and their mechanisms, which primarily focus on ion channel modulators, analgesics, neuro-immune/metabolic modulators, anti-AGEs, and neurotrophic or regenerative medicines (Table 2).

**Table 2:** Drugs and biotherapy in clinical trials worldwide (since 2020).

Treatments	Mechanism		Clinical trial number	Phase	Related Ref
Pregabalin extended-release tablets	Ion channel modulator	α_2_δ subunit of VGCCs	NCT06383702	Phase 3	[112]
HSK16149		α_2_δ subunit of VGCCs	NCT06490484	Phase 2	[113]
DS-5565		α_2_δ subunit of VGCCs	NCT02318706	Phase 3	[3,114]
Suzetrigine		NaV1.8 inhibition	NCT06696443	Phase 3	[116]
VX-993		NaV1.8 inhibition	NCT06619860	Phase 2	[117]
VX-150		NaV1.8 inhibition	NCT03304522	Phase 2	[118]
BIIB074		Nav1.7 selective sodium channel blocker	NCT03339336	Phase 2	[119]
LY3857210		P2X7 receptor antagonist inhibits calcium influx in cells expressing P2X7 receptors	NCT05620576	Phase 2	[120]
Eliapixant		Selective P2X3 antagonism	NCT04641273	Phase 2	[121]
LY3556050		Somatostatin receptor 4 (SSTR4) agonists affect potassium and calcium currents	NCT06074562	Phase 2	[122]
NYX-2925		NMDAR modulator	NCT04146896	Phase 2	[123]
MT-8554	Analgesics	NK3R antagonists	NCT05123196	Phase 2	[125]
Tanezumab		Analgesics, anti-NGF antibody blocks NGF-mediated neuronal sensitization	NCT01087203	Phase 2	[126]
BAY2395840		Analgesics, bradykinin B1 inhibitor blocks EGFR-mediated neuronal sensitization	NCT05219812	Phase 2	[127]
YJ001		Analgesics	NCT06361108	Phase 1	[128]
THC inhaled		Pain relief through cannabinoid receptors (CB1 and CB2)	NCT06490445	Phase 2	[129]
AP707		Pain relief through regulating endocannabinoids	NCT06072573	Phase 3	[130]
CBME		Reactive with CB1 and CB2 for antioxidant activity and neurotrophic effects	NCT00238550	Phase 2	[131]
Incobotulinumtoxin-A	Neuro-Immune/Metabolic Modulators	Downregulation of TRPV1 and sodium channels, anti-inflammatory, inhibition of pain-related neurotransmitters	NCT05623111	Phase 2	[132,133]
			NCT05296759	Phase 4	
Combination of alpha lipoic acid and vitamin B		Antioxidant, anti-inflammation, metabolic regulator, and neurotrophic	NCT06568185	Phase 2	[134]
Vitamin D3		Neurotrophic factor support and anti-inflammation	NCT04984044	Not Applicable	[135]
Melatonin		An agonist of melatonin receptors (MT1 and MT2); antioxidant, anti-inflammatory, and upregulates NRF2	NCT07036796	Phase 2	[136]
Nebivolol		β-blocker, reduce oxidative stress and regulate metabolism	NCT06201611	Phase 3	[137]
Semaglutide		GLP-1 receptor agonists, antioxidant, and anti-inflammation	NCT06461377	Phase 4	[138]
Piracetam		An allosteric modulator of receptors protects against oxidative stress and inflammation	NCT06479629	Phase 4	[139]
LX9211		Pain relief by inhibiting the adaptor-associated kinase 1 (AAK1) enzyme	NCT04455633	Phase 2	[140]
Ricolinostat		Histone deacetylase 6 inhibitor, anti-inflammation, and mitochondria improvement	NCT03176472	Phase 2	[141]
Cytoflavin (Inosine + nicotinamide + riboflavin + succinic acid)		Antioxidant activity	NCT04649203	Phase 3	[142]
Resveratrol, alpha lipoic acid, superoxide dismutase		Antioxidant activity	NCT06131918	Phase 2	[143]
Canagliflozin		SGLT2 inhibitor, anti-inflammation, and blood sugar regulation	NCT02065791	Phase 3	[3,145]
Empagliflozin			NCT05977465	Phase 2	[3,146]
Forxiga			NCT04193566	Phase 4	[147]
EMA401			NCT03094195	Phase 2	[148]
Pirenzepine		Muscarinic receptor antagonists alleviate peripheral and central sensitization, axon growth	NCT04786340	Phase 2	[149]
Roflumilast		PDE4 inhibitor, anti-inflammation	NCT05369793	Phase 3	[144]
Pyridoxamine Supplementation	Anti-AGE	Scavenges methylglyoxal, thereby inhibiting the formation of AGE	NCT06376240	Not applicable	[150]
Benfotiamine		Anti-AGEs, antioxidant	DRKS00014832	Phase 2	[3,151]
Autologous bone marrow-derived stem cells (BMSC)	Neurotrophic & Regenerative	Anti-inflammation, angiogenesis and neurotrophic support	NCT02795052	Not Applicable	[152]
Acetyllevocarnitine hydrochloride tablets		Stimulate nerve regeneration and provide neuroprotection	NCT05319275	Phase 3	[153]
VM202		Express two HGF isoforms, cHGF and dHGF, with neurotrophic and angiogenic activities	NCT02427464	Phase 3	[154]

Ion channel modulators and analgesics aim to relieve pain through modulating neuronal excitability. Pregabalin extended-release tablets, HSK16149, and DS-5565 alleviated pain by targeting the α_2_δ subunit of voltage-gated calcium channels (VGCCs), affecting neuronal excitability and neurotransmitter release [3,[112], [113], [114]]. NaV1.7 and NaV1.8 are generally involved in pain initiation and maintenance, respectively [115]. NaV1.7 inhibitor (BIIB074) and NaV1.8 inhibitors (Suzetrigine, VX-993, VX-150) effectively relieved trigeminal and neuropathic pain, respectively [[116], [117], [118], [119]]. Purinergic receptor X7 (P2X7) and Purinergic receptor X3 (P2X3) antagonists (LY3857210, Eliapixant) blocked calcium influx and then achieved pain relief [120,121]. LY3556050 targets somatostatin receptor 4 (SSTR4), affecting both potassium and calcium currents to relieve pain [122]. NYX-2925ZE modulated the ionotropic glutamate receptor N-methyl-D-aspartate (NMDA) receptor to regulate neuronal excitability, thereby relieving pain [123,124]. The analgesic drug MT-8554 directly relieved trigeminal neuralgia by targeting the neurokinin 3 receptor (NK3R) [125]. Tanezumab and BAY2395840 targeted neuronal growth factor (NGF) and epidermal growth factor receptor (EGFR) signaling, respectively, to suppress neuronal sensitization [126,127]. YJ001 is a spray-type analgesic that relieves pain by topical application to the foot skin [128]. Cannabinoids such as Delta (Δ)9-tetrahydrocannabinol (THC) inhaled, AP707, and Cannabis-based medicine extract (CBME) achieve analgesia by interacting with cannabinoid receptors 1 (CB1) and 2 (CB2) [[129], [130], [131]].

Neuro-immune and metabolic regulators primarily improve the DPN microenvironment by modulating antioxidant activity, inflammation, and metabolism. Incobotulinumtoxin-A alleviated pain by downregulating TRPV1, sodium channels, pro-inflammatory effects, and inhibition of pain-related neurotransmitters [132,133]. Combined α-lipoic acid and vitamin B therapy exerted antioxidant, anti-inflammatory, metabolic-regulatory, and neurotrophic effects, promoting regeneration while relieving pain [134]. Vitamin D3 [135], melatonin, an agonist of Metallothionein 1 and 2 (MT1, MT2) and nuclear factor erythroid 2-related factor 2 (NRF2) [136], nebivolol (a β-blocker) [137], and semaglutide (glucagon-like peptide-1 receptor agonist, GLP-1RA) [138] provide neuroprotection and pain relief through anti-inflammatory and metabolic pathways. Piracetam [139], LX9211 (adaptor-associated kinase 1 inhibitor) [140], ricolinostat (histone deacetylase 6 inhibitor) [141], cytoflavin [142], a mixture of resveratrol, alpha lipoic acid, and superoxide dismutase [143], BAY2395840 (bradykinin b1 inhibitor) [127], and roflumilast (phosphodiesterase-4 inhibitor) [144] relieved pain through reducing inflammation or oxidative stress. Canagliflozin, Empagliflozin, Forxiga, and EMA401 act as sodium-glucose cotransporter 2 (SGLT2) inhibitors to alleviate pain progression through anti-inflammatory and glucose regulation [3,[145], [146], [147], [148]]. Pirenzepine promotes axonal growth by interacting with muscarinic receptors, alleviating peripheral and central sensitization [149].

AGE-related aging can promote the development of DPN. Pyridoxamine supplementation and benfotiamine, as anti-AGE drugs, inhibited AGE formation and thus controlled the progression of DPN [3,150,151]. Neurotrophic and regenerative therapies are also developing. Autologous bone marrow-derived stem cells (BMSCs) promoted nerve regeneration and pain relief through anti-inflammatory, angiogenesis, and neurotrophic support [152]. Acetyl levocarnitine hydrochloride tablets inhibited DPN progression by supporting neuroprotection and regeneration [153]. VM202, a gene-targeted drug, promoted the expression of two HGF (hepatocyte growth factor) isoforms (cHGF and dHGF) to promote nerve regeneration and analgesia [154].

Overall, current drugs in preclinical research aim to relieve pain or promote nerve regeneration by targeting key pathological mechanisms [3]. Although these candidates are promising, further validation through assessment of the side effects and large-scale double-blind clinical trials is essential to verify their efficacy and feasibility [3,61].

#### 3.2.3 Biomaterials-based drug delivery system

To enhance drug therapeutic efficacy and reduce adverse effects, biomaterial-based drug delivery systems have been widely applied in the biomedical field. Their advantages include precise targeting, tissue-specific delivery, sustained release, preservation of active ingredients, reduced systemic toxicity, and synergistic treatment for enhanced efficacy [5,155].

**Fig. 3:**
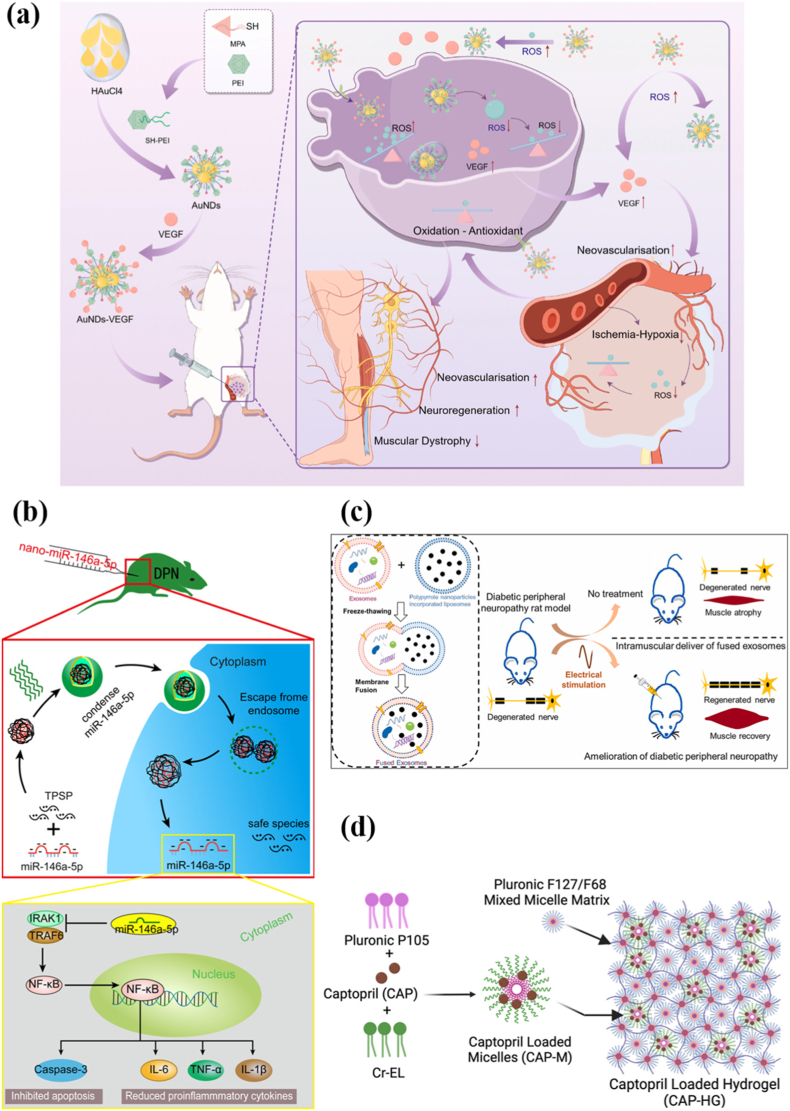
(a) The mechanism of VEGF-loaded ROS-responsive nanodots to improve sciatic neuropathy in diabetic peripheral neuropathy [161]. (b) The mode chart of nano-miR-146a-5p transfection and the potential pathway of miR-146a-5p inhibition of the inflammatory response and apoptosis [163]. (c) Transplantation of engineered exosomes derived from bone marrow mesenchymal stromal cells ameliorated diabetic peripheral neuropathy under electrical stimulation [164]. (d) Synthetic scheme for captopril micelle (CAP-M) formation and incorporation into the mixed Pluronics (Pluronic F127/F68) [168].

Nanoscale delivery systems have demonstrated promising applications in pain management, offering higher efficacy, lower analgesic doses, and prolonged pain relief [5]. Research using nanomaterials to improve DPN has been extensively explored. Emulsified nanoparticles have improved the solubility, dissolution, and absorption of poorly soluble drugs. Curcumin-encapsulated PEG and poly-PEGMA-DMAEMA-MAMMAM nanoparticles reduced neuroinflammation, enhanced antioxidant defenses, and suppressed excitatory transmission of P2X3 receptors in the dorsal root ganglion (DRG) of T2DM rats, reversing the sensorimotor and biochemical deficits caused by DPN [156,157]. Similarly, nanoemulsion-based delivery of capsaicin enhanced skin penetration and alleviated pain in mice with DPN [158]. Hafiz et al. prepared nano-ethosomes that delivered quercetin borate, epalrestat, and urea, which successfully alleviated pain in DPN rats by promoting antioxidant activity [159]. Metal-based nanoparticles have also been employed. Silver nanoparticles combined with *Nigella sativa* extract exhibited anti-diabetic, anti-inflammatory, and antioxidant effects [160]. Additionally, ROS-responsive thiol-modified gold nanoparticles grafted with VEGF successfully demonstrated antioxidant and angiogenic, and sciatic nerve regeneration in a rat DPN model (Fig. 3a) [161]. Yao et al. used tetrahedral backbone nucleic acids (tFNAs) loaded with resveratrol (RSV) to improve redox regulation and energy metabolism in mice DPN, restoring sensory function and promoting neurovascular regeneration [162]. Qiong et al. addressed the progression of rat DPN by regulating inflammatory responses using a cationic nanocarrier imine backbone polymer (TPSP) loaded with miRNA-146a-5p delivery system (Fig. 3b) [163]. Anamika et al. fused exosomes derived from BMSCs with liposome-containing polypyrrole nanoparticles (PpyNps), combining both biophysical cues and exogenous electrical stimulation to promote nerve regeneration (Fig. 3c) [164].

Macro- and micro-scale delivery systems, such as dressings, injectable hydrogels, and microneedle patches, enable sustained, localized drug release when applied to the skin and nerves. For example, Zhou et al. developed a novel pH-responsive conductive hydrogel of poly (3,4-ethylenedioxythiophene) polystyrene sulfonate (PEDOT: PSS) loaded with salidroside-derived carbon dots (CDs) and magnesium silicate nanoflowers [165]. Intramuscular injections of this system inhibited Schwann cell pyroptosis and promoted neurovascular remodeling by scavenging reactive oxygen species and restoring endogenous electric fields through CDs and Mg^2+^ [165]. Androschuk et al. applied methacrylic acid (MAA)-based hydrogels to increase the expression of key neuronal markers and growth factors, enhancing nerve regeneration in DPN mice [166]. Thermosensitive hydrogels delivering bFGF, NGF, or angiotensin II (Ang II) have also promoted nerve regeneration and pain relief (Fig. 3d) [167,168]. Microneedles (MNs) are widely used in transdermal drug delivery applications due to their non-invasive, painless, and controlled release properties [169]. Wang et al. designed a segmented microneedle for DPN treatment. Silk fibroin hydrogel formed the microneedle tips with continuous release of vitamin B9, while the chitosan/polyvinyl alcohol (PVA) base was functionalized with boronate bonds, releasing insulin in response to glucose fluctuations [170]. Additionally, given that DPN increases susceptibility to nerve compression, Singh et al. used BMSC exosomes with nerve conduits to repair traumatic nerve injury in a rat DPN model [171].

Despite the remarkable progress, several challenges remain that need to be clarified (Fig. 2). 1) Clinical translation: Existing FDA-approved or preclinical-stage drugs exhibit dose-dependent side effects [61]. It is necessary to explore appropriate biomaterial delivery systems to achieve controlled release and reduce systemic toxicity. 2) Lack of disease stage-specific targeting: DPN progresses through early stage (onset of metabolic disorders, neurological function changed), mid-stage (structural damage), and late stage (irreversible neurodegeneration) [71]. Current delivery strategies lack temporal specificity; for example, antioxidant therapy in the early stage, whereas neuroregeneration cues in the late stage. 3) Systemic metabolic coordination: Complex systemic metabolic dysregulation appears in DPN. There is an urgent need to develop multimodal delivery systems that coordinate the neural microenvironment and systemic metabolism. 4) Neglect of nerve interactions in diabetic wounds: Diabetic wounds or ulcers are often accompanied by DPN, yet most wound repair materials (such as antibacterial hydrogels) ignore nerve repair assessment (such as nerve regeneration, behavior, etc.), especially relevant given the frequent use of STZ-induced diabetic models across both domains [[172], [173], [174], [175], [176], [177]]. 5) Limited animal model diversity: Current animal models used for biomaterial delivery research are mainly STZ- or ALX-induced mice [[156], [157], [158],^160^,^161^,[163], [164], [165],^167^,^170^,^171^], with few employing genetic models (e.g., db/db or Leprdb mice) [162,166,168]. Given the diverse etiology of DPN, broader model utilization, including the ZDF model (obesity-related) or the SHR model (neurovascular interactions), is needed [[100], [101], [102], [103]]. Furthermore, the exploration of large animal models for DPN delivery system research remains limited.

## 4 Microfluidic platforms for *in vitro* modeling and therapeutic research

Microfluidic platforms are emerging tools in biomedical applications. They are characterized by engineering and manipulating fluids at the submillimeter scale and have shown great potential in biological research [[178], [179], [180]]. Although traditional 2D cell culture techniques have been widely used in biomedical research, they often fail to recapitulate the tissue-specific characteristics and the physiological microenvironment necessary for accurately predicting *in vivo* tissue function and drug activity [181,182]. To address these limitations, 3D cell culture models have been developed, including the hanging drop, forced floating, matrix/scaffold, stirring, and microfluidics [183]. Compared with other methods, the microfluidics method can easily create chemical/nutrient and oxygen concentration gradients, spatial distribution of different cell types (including tissue-tissue interfaces), and mechanical cues to simulate complex and dynamic 3D networks *in vivo* [184,185]. These features enable the construction of organ-on-a-chip (OoC) models that integrate multiple tissue types and microenvironmental signals to more closely replicate *in vivo* physiology. Neurobiologists have used microfluidic platforms to study various aspects of the nervous system, such as axonal function, synapse formation and function, interaction between neurons and different cells, and the spatial movement behavior of glial cells [[186], [187], [188], [189]]. Moreover, various neurological disease models have been established for research. Multiple types of nerve injuries (such as compression, stretching, and transection) can be reproduced using microfluidic devices, and cell behaviors (such as axon regeneration) can be studied at the molecular level [6]. Given that the intrinsic mechanisms underlying neurodegenerative diseases (such as Alzheimer's disease, Parkinson's disease, and amyotrophic lateral sclerosis) and neurometabolic diseases (such as DPN) remain unclear, and microfluidic platforms can serve as the first line of testing for biological mechanism research and drug development to reduce animal testing steps [190,191]. Herein, we review the construction of *in vitro* DPN models and highlight the microfluidic platforms currently used for DPN therapeutic research.

### 4.1 Microfluidic *in vitro* models for DPN

**Fig. 4:**
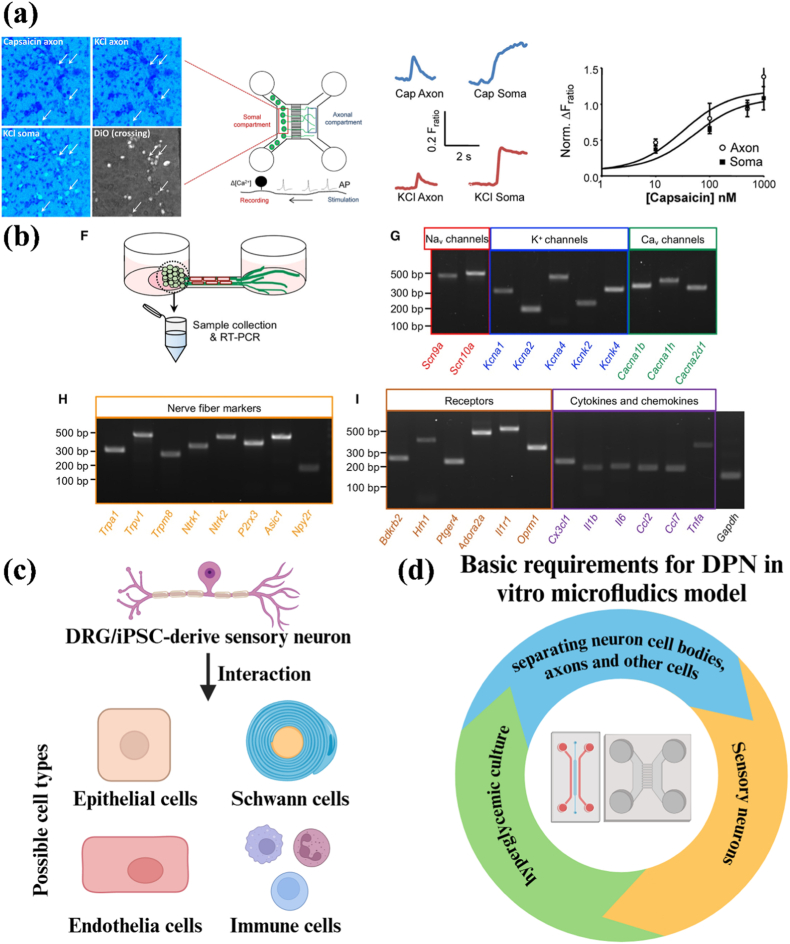
a) Transduction and transmission of stimuli by axons in Microfluidic chambers (MFCs) [192]; b) Structural characteristics and ganglion expression of representative ion channels, receptors, and sensory neuron markers in the sensory nerve organotypic model: Structural characteristics and ganglion expression of representative ion channels, receptors, and sensory neuron markers in the sensory nerve organotypic model: After 28 days in culture, the ganglion-like structures in the microfluidic-based organotypic model successfully expressed key ion channels, including voltage-gated sodium and potassium channels that facilitate action potential initiation and propagation. They also showed expression of N-type and T-type calcium channels involved in pain signaling, along with the α2δ subunit (Cacna2d1), a target for gabapentin therapy. Additionally, the model expressed neurotrophin receptors, C-fiber markers such as transient receptor potential (TRP) channels and P2X3 (P2r), and receptors linked to inflammation, such as bradykinin receptor B2 (Bdkrb2) and histamine receptor H1 (Hrh1). mRNA levels of proinflammatory cytokines and chemokines were also observed. [98]; c) Potential cell types for reproducing the interaction between sensory neurons and PNS, ceated by Biorender; d) Basic requirements for DPN *in vitro* microfluidics model, created by Biorender.

Microfluidic models used to study the peripheral nervous system (PNS) typically have multiple interconnected or independent chambers that separate neuronal cell bodies, axonal regions, or other cell types [190]. The design enables precise local stimulation and independent control of the environment to mimic the anatomical structure of the PNS. Since the most common clinical manifestation of DPN is neuropathic pain, DRG has become the most used cell in microfluidic platforms to reproduce the function of axons *in vitro*. For instance, Christoforos et al. evaluated the function of DRG cultured in microfluidic chips using calcium imaging (Fig. 4a). Stimulation of DRG axons resulted in local depolarization (like stimulation of nociceptor nerve endings *in vivo*) and the generation of action potentials (APs) that propagated toward the cell body. When these APs reached the cell body, they triggered the opening of voltage-dependent calcium channels. After stimulation of the axons with capsaicin (100 nM) or KCl (30 mM), the DRG cell body was activated, resulting in a further increase in intracellular Ca^2+^ and the generation of detectable signals [192]. Madoka et al. successfully expressed ion channels, receptors, cytokines, and chemokines that are essential for sensory nerve function in DRG *in vitro* (Fig. 4b) [98]. To further simulate the PNS microenvironment, other cell types can be introduced into the model, including Schwann cells, epithelial cells, endothelial cells, and immune cells (Fig. 4c) [190,[193], [194], [195]]. Moreover, to recapitulate the microenvironment of DPN, the hyperglycemic concentration in the culture medium is regulated in the *in vitro* culture system to simulate the diabetic state. Under hyperglycemic conditions, cells produce oxidative stress, inflammatory factors, and other metabolites, thereby altering the cell microenvironment [[194], [195], [196], [197]]. Therefore, the basic requirements for constructing a DPN microfluidics model are: 1) compartmentalization of neuronal cell bodies, axons, and other cells; 2) use of sensory neuronal cell bodies; 3) hyperglycemic culture environment (Fig. 4d).

### 4.2 Therapeutic research for DPN: mechanisms and drug development

The microfluidic chips currently most widely used for DPN therapeutic research are conducted under hyperglycemic conditions. The microfluidic chip consists of two chambers: one for seeding neuron somas, another for other cell types, or for providing space for axon aggregation, with an intermediate zone for axon growth. Since Schwann cells are the predominant myelinating cells in the PNS and are vulnerable to hyperglycemic damage, their impairment can adversely affect DRG function. Argonaute2 (Ago2) is a key molecular participant in mediating miRNA-guided mRNA cleavage activity and miRNA stability. Fan et al. cultured DRG isolated from Schwann cells-Ago2-KO mice with DPN and DRG isolated from WT mice with DPN on a microfluidic chip to observe axon growth. They found that axon growth in the Ago2-KO group was significantly reduced. The underlying mechanism appeared to involve the regulation of Ago2 in the RAGE/NF-κB pathway, which affected the mitochondrial function of Schwann cells by changing the miR-206 binding in peripheral nerves [198]. In another study, Jia et al. co-incubated exosomes derived from hyperglycemic Schwann cells and DRGs under normal glucose conditions on a microfluidic chip. They found that exosomes secreted by HG (high glucose)-treated Schwann cells inhibited axon growth. The possible mechanism was that increased levels of miR-28, -31a, and −130a inhibited axon growth; this finding was also validated *in vivo* [194]. Additionally, they studied changes in the DRG itself under hyperglycemic conditions. The results showed that miR-34a and its target genes, forkhead box protein P2 (FOXP2) and vesicle amine transport 1 (VAT1), were involved in DRG damage under hyperglycemia, resulting in reduced axon growth [196]. Moreover, their results also showed that miR-29c acted as a negative regulator of DRG axon growth under hyperglycemia by targeting Atypical PKCι/λ [199]. These findings suggest that targeting specific microRNAs may be a potential therapeutic approach to alleviating the progression of DPN.

**Fig. 5:**
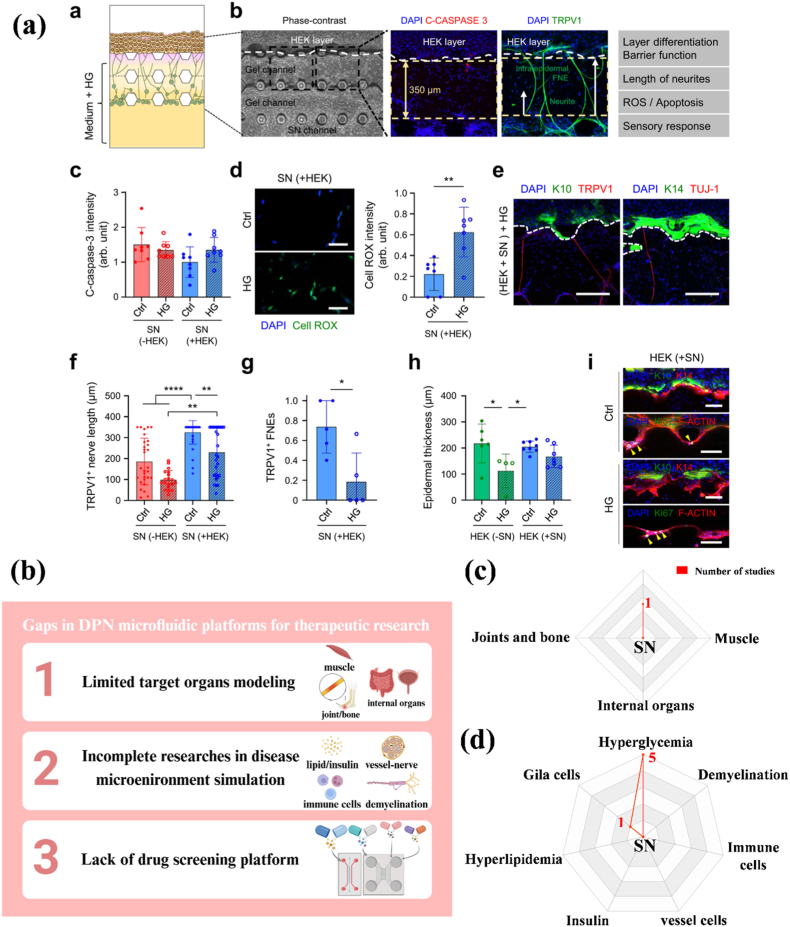
a) Acute hyperglycemia-induced pathological modeling using innervated epidermal-like layer chips: The study examined high glucose effects on the substantia nigra's survival, apoptosis, and oxidative stress by analyzing caspase-3 markers and ROS levels. Results showed increased ROS causes oxidative stress. While the number of TRPV1+ neurons didn't change, nerve fibers in the epidermis decreased in length and number. Axonal growth and innervation were specifically suppressed. High glucose didn't significantly affect apoptosis, proliferation, or epidermis thickness. [195]; b) Schematic of gaps in DPN microfluidic platforms for therapeutic research, created by Biorender; c) Statistical analysis of research trends on target organs innervated by sensory neurons (SN); d) Statistical analysis of research trends of SN in reproducing the DPN disease microenvironment.

The study of DRG and its target organs in DPN should also be taken seriously. In particular, dysfunction of intraepidermal nerve fibers in response to changes in the skin microenvironment in DPN may play an important role. Ahn et al. constructed a microfluidic platform to investigate the pathophysiological mechanisms underlying intraepidermal nerve fibers and epidermal keratinocytes in the development and progression of hyperglycemia-induced neuropathy (Fig. 5a) [195]. Obvious oxidative stress (ROS accumulation) appeared in sensory nerves (SN) under HG, with marked reductions in axon length and number, leading to impaired axonal growth and epidermal innervation. It is worth noting that when capsaicin was topically applied to the innervated epidermal layer, a Ca^2+^ influx reaction occurred in epidermal neurons at the single-cell level. Although hyperglycemia reduced intraepidermal SN numbers, afferent transmission from epidermal keratinocytes to SN increased slightly. The results indicated that the microfluidic platform successfully simulated the oxidative stress state of diabetic patients and reproduced a skin state susceptible to infection due to barrier defects (changes in material permeability), providing a potential mechanism for the early features of acute hyperglycemia or prediabetes [195].

In general, current microfluidic platforms based on DPN successfully simulate the hyperglycemic microenvironment and provide valuable insights into the molecular mechanism and potential therapeutics for DPN (Table 3). However, there are still several gaps remaining in this field (Fig. 5b): 1) Limited target organs modeling: The target organs innervated by DRG include not only the skin but also skeletal muscles, joints, and bones, as well as some internal organs [200,201]. However, effective *in vitro* microfluidic platforms to the study sensory neurons along with their target organs are lacking (Fig. 5c); 2) Incomplete simulation of the disease microenvironment: From the mechanistic/pathophysiological perspective, current *in vitro* microfluidic platforms do not fully replicate key aspects of DPN pathophysiology beyond hyperglycemia, including hyperlipidemia, insulin dysregulation, microvessels changes (the DPN neurovascular interface), inflammation (immune cells or inflammatory molecules), and demyelination [21,202] (Fig. 5d); 3) Lack of drug screening platform: Although several platforms have been applied for diabetes drug screening, no drug screening system dedicated to DPN exists [203,204]. The DPN model drug screening system should also emphasize the neuronal component. Furthermore, while microfluidic systems show great potential for reducing reliance on animal models, studies of pain or behavioral outcomes still require animal experiments. Taken together, both *in vitro* environmental simulations and their applications often lack the inclusion of multiple target tissues, such as skin, muscle, and bone, along with DRG and DPN microenvironmental signals. Future progress should focus on adopting the OoC approach by integrating diverse tissues and microenvironmental cues into a single platform to create a DPN-on-a-chip. This would enable more physiologically relevant models, facilitate mechanistic studies, and support *in vitro* drug screening.

**Table 3:** Microfluidic platforms for DPN therapeutic research.

Platform design	Cell types	Key findings	Ref
Local stimulation of DRG in hyperglycemia	DRG	HG exposure impaired axonal regeneration through mir-28, 31a, and 130a	[194]
3D bionic chip with multi-chamber design for division and gradient formation of DRG and keratinocytes	DRG, keratinocytes	Reconstruction of barrier defects similar to the nerve-skin interface *in vivo* under DPN	[195]
Microfluidic system for localized HG exposure for molecular mechanism studies	DRG	Dysregulation of miR-34a led to impaired axonal growth by targeting FOXP2 and VAT1	[196]
Microfluidic co-culture system with controlled local microenvironment	DRG, SCs	HG disrupted SCs function and impairs DRG axon growth	[198]
Microfluidic system for localized HG exposure for molecular mechanism studies	DRG	Identification of miR-29c as mediating axonal growth inhibition under HG exposure	[199]

## 5 Bioengineered devices for DPN assessment and monitoring

From a clinical perspective, assessment and monitoring are as important as therapeutic interventions throughout the entire progression of DPN. In the early stages, many people with diabetes remain without symptoms until nerve damage becomes severe [205]. Thus, systematic and early detection of small fiber dysfunction can prevent irreversible nerve damage, aiding in risk stratification and preventive strategies [206]. In diagnosed and advanced DPN cases, assessment and monitoring remain crucial because the condition is often progressive and varies among individuals [207]. Ongoing monitoring helps track disease progression, assess treatment effectiveness, and identify complications like sensory loss and gait issues [208]. Since no current treatment can fully reverse DPN [3], this emphasizes the importance of regular assessment and monitoring.

Bioengineered devices for DPN assessment and monitoring are designed to measure or visualize pathophysiological changes, thereby providing objective, reproducible data. These bioengineering devices can be divided into two categories: 1) commercial DPN assessment devices for scoring, diagnosis, evaluation, and classification; and 2) emerging DPN monitoring technologies with AI-driven approaches and wearable devices. Most commercially available DPN assessment devices are still limited to static evaluations, such as capturing disease status at a single time point. These assessments offer limited insight into disease dynamics, progression, treatment response, or individual variability. Emerging technologies aim to enable earlier, continuous, specific, and personalized monitoring to improve risk stratification and dynamic assessment, marking a shift from static evaluation to proactive disease management. Collectively, these bioengineered devices represent the current landscape of available and promising methods for DPN assessment and monitoring, bridging the gap between research innovations and bedside applications.

### 5.1 Commercialized DPN assessment devices

Commercialized DPN assessment devices have been widely used in translating clinical and research findings to practical tools for screening, diagnosis, and disease progression monitoring. Current commercialized devices can be broadly categorized into four groups: 1) preliminary assessment devices provide rapid bedside screening; 2) electrophysiological assessment devices measure the nerve conduction and excitability; 3) physiological assessment devices test the biomechanical or sensory function; 4) image assessment devices visualize the peripheral nerve structure or changes in microvascular.

#### 5.1.1 Preliminary assessment devices

**Fig. 6:**
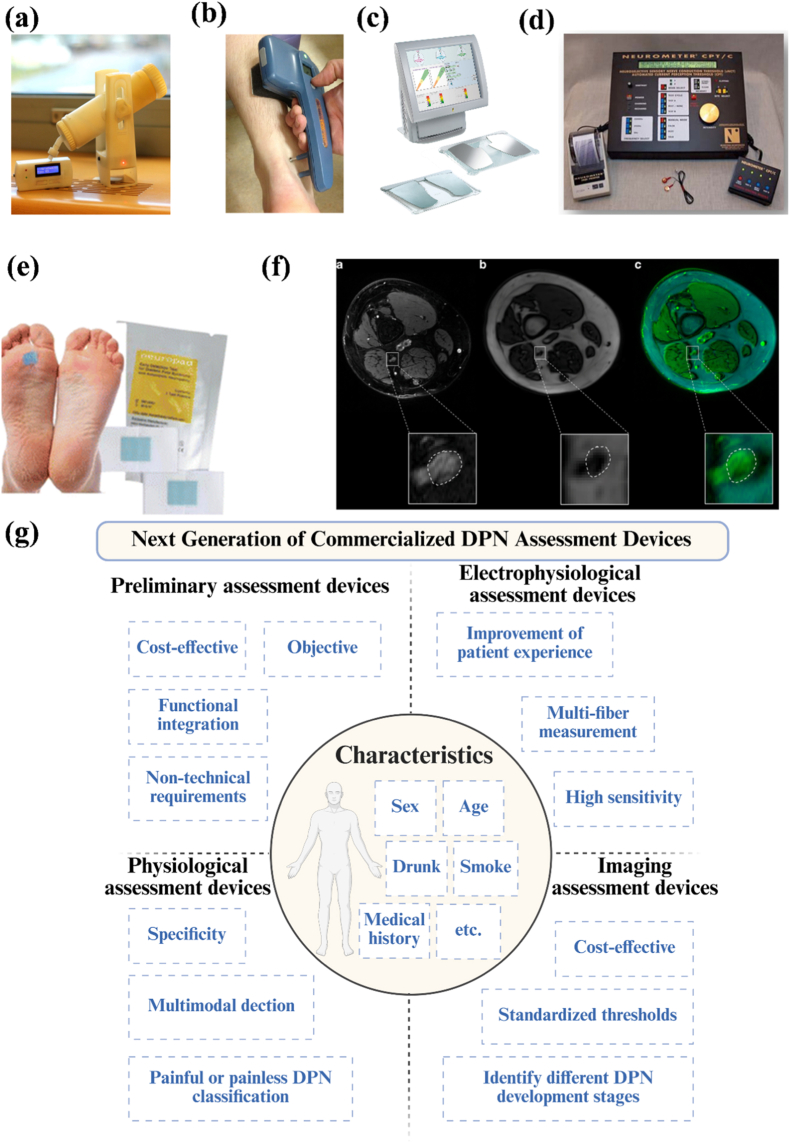
a) NerveCheck is a portable, inexpensive ($500) quantitative sensory device designed to assess vibration (VPT), cold (CPT), warm (WPT) perception threshold, and heat pain threshold [216]; b) the point-of-care procedure of DPNCheck™ [228]; c) Sudoscan device with hand and foot electrodes [238]; d) The Neurometer® device [241]; e) The Neuropad device [271]; f) The process of coregistration of MRN sequences of a patient with T2D and small fiber neuropathy [294]; g) Next generation of commercialized DPN assessment devices, created by Biorender.

Initial screening for DPN is performed by providing feedback to the patient after stimulation. Currently, the technologies used for clinical testing include vibration, pressure, noxious stimulation (pinprick), and heat/cold stimulation [7]. Standard devices for generating vibration were traditional tuning forks, electronic tuning forks, and mobile phones [209,210]. Pressure perception is commonly assessed using a 5.07/10 g Semmes-Weinstein monofilament to assess skin pressure perception [209,211]. Digital biovibrometers can also be used for pressure measurement, with the probe applying steady pressure perpendicular to the test site [212,213]. For noxious stimulation (pinprick), a sterile pin, toothpick, or cotton swab is used to touch the patient's foot [7]. Heat/cold stimulation is performed by placing a temperature test pen on the back of the foot at irregular intervals to assess the patient's temperature perception function, or by eliciting a thermomotor response on the skin [7,214,215]. NerveCheck is the first affordable, portable device for quantifying sensory loss that performs both vibration and heat testing simultaneously (Fig. 6a) [216]. Each device provides unique information, and compared with using them individually, combining them with an integrated assessment system offers a more comprehensive assessment of DPN clinical symptoms.

Common comprehensive DPN assessment systems based on various initial screening devices include the Michigan Neuropathy Screening Instrument (MNSI), Toronto Clinical Scoring System (TCSS), Neuropathy Disability Score (NDS), and quantitative sensory testing (QST) [[217], [218], [219], [220], [221]]. Table 4 summarizes these common comprehensive assessment systems. These devices offer the advantages of simplicity, low cost, and high standardization, making them suitable for large-scale screening and monitoring disease progression. However, they are significantly influenced by patient subjective factors, and some devices may lack sufficient sensitivity or specificity, thus affecting the final diagnostic outcome. Regarding new preliminary screening devices, proposals should be cost-effective, objective, and functionally integrated. For preliminary screening across different populations, clinical data can serve as a reference (Fig. 6g).

**Table 4:** Main scoring systems for DPN assessment.

Scoring system	Assessment devices	Scoring criteria	Advantages	Limitations	Ref
Michigan Neuropathy Screening Instrument (MNSI)	Vibration-generating devices, monofilament testing	Evaluate appearance, tingling, numbness, burning sensation, etc. The risk of DPN is evaluated by the scores of questionnaires and tests	Widely used, low-cost, simple, and fast management	Subjectivity is strong, and different testers affect the consistency of results	[217]
Toronto Clinical Scoring System (TCSS)	Devices for vibration generating, thermal variation, pinprick, and reflexes	Evaluate foot pain, numbness, tingling, weakness, ataxia, and upper limb symptoms. The severity of DPN is categorized from 0 to 3 based on symptoms and tests	Comprehensive evaluation and standardization of DPN severity grading	Strong subjectivity and time-consuming	[220]
Neuropathy Disability Score (NDS)	Devices for vibration, generating, thermal variation, and ankle reflex testing	Score the test results, and the sum of the scores represents whether there is an abnormality in nerve function	Widely used in clinical, low-cost, simple, and fast pre-screening and monitoring	Strong subjectivity and may not be sensitive to early symptoms	[218]
Quantitative Sensory Testing (QST)	Devices for testing the perception threshold of different stimuli (such as temperature, vibration, pressure, etc.)	Sensory thresholds are assessed for each test to determine sensory loss or hyperactivity	Provide an objective and quantitative sensory function assessment that can detect early DPN	Requires special equipment and professionals, time-consuming and costly	[221]

#### 5.1.2 Electrophysiological assessment Devices

Electrophysiological assessment is the gold standard for the diagnosis of peripheral neuropathy and serves as the minimal non-invasive measure of the condition [217,222]. The earliest clinical manifestations of DPN are related to lesions of all primary sensory fibers: large-diameter myelinated fibers Aβ, small thinly myelinated Aδ fibers, and unmyelinated C fibers [206]. Additionally, the CNS receives abnormal pain signals and is damaged after DPN [223]. Therefore, the equipment used for electrophysiological assessment of DPN is usually targeted at sensory fibers and the CNS.

Nerve conduction studies (NCS) have long been used as a diagnostic, staging, and prognostic marker test for DPN. In most cases, electrodes were placed on the median, ulnar, peroneal, tibial, and sural nerves to test the function of Aβ fiber [224,225]. NCS was often used in conjunction with electromyography (EMG) to assess the neuromuscular system [226]. However, NCS requires expensive equipment and highly trained technicians [227]. A new point-of-care diagnostic nerve conduction device, the DPNCheck™ device, is suitable for non-technical personnel (Fig. 6b) and has shown high accuracy compared with NCS [228,229].

A more sensitive test for DPN is to assess small-diameter fibers since they are damaged earlier than large-diameter fibers [206]. Evoked potentials (EP) have been considered a non-invasive and reliable tool for studying the function of Aδ and C fibers. EP is divided into laser-evoked potentials (LEP) and contact heat-evoked potentials (CHEP) [230,231]. To generate heat stimulation, LEP used a CO_2_ laser [231], and CHEP used a heat stimulator placed on the skin [232]. Finally, the signals generated by LEP or CHEP were collected by electroencephalography (EEG) to assess the progression of DPN [233].

EEG can also be used as a standalone device to determine the patient's pain response. EEG signals can be divided into multiple frequency bands (such as δ, θ, α, β, γ bands) to analyze the CNS, which MRI cannot do [234]. Each frequency band is associated with specific neural activity and cognitive functions and analyzing these frequency bands reveals the function of the brain in different states [235]. Using Standardized Low-Resolution Electromagnetic Tomography (sLORETA) to convert discrete electrode data into continuous brain activity maps and then apply voxel-based analysis, the results distinguished between chronic pain DPN patients and control subjects [234,236].

Sweat gland dysfunction is one of the earliest detectable neurophysiological abnormalities in distal small fiber neuropathy [237]. Sudoscan is an FDA-approved device that measures electrochemical skin conductance (ESC) to assess the function of the sweat gland (Fig. 6c) [206,238]. The current generated is proportional to the chloride ion concentration to which the electrodes react, and a low ESC indicated abnormal sweat gland function [238]. The test takes less than 3 min to complete and required no special patient preparation or medical staff training [239].

Neurometer® is a well-established, painless, transcutaneous peripheral nervous system current perception threshold (CPT) test device that, compared with other electrophysiological assessment tools, can simultaneously assess Aδ and C fibers [240]. The Neurometer® used electrodes on the fingers, toes, or trigeminal nerve to generate transcutaneous electrical stimulation at frequencies of 2000 Hz (Aβ fibers), 250 Hz (Aδ fibers), and 5 Hz (C fibers) to assess the three subtypes of nerve fibers (Fig. 6d) [240,241]. Existing clinical data supported that Neurometer® diagnosed early DPN in both T1DM and T2DM and demonstrated higher accuracy than the monofilament testing method [241,242].

Microneurography involves inserting a fine tungsten electrode into a peripheral nerve via a minimally invasive approach to record activity from the common or superficial peroneal nerve, thereby helping evaluate neuropathy [243]. The potential difference between the tungsten electrode and a reference electrode is measured in millivolts and reflects neural activity [244]. This technique enables simultaneous recording of multiple C-fibers and the identification of different C-fiber subtypes [245]. In patients with painful DPN, there is an increased proportion of more excitable, sensitized, and mechanically insensitive C-fibers, along with changes in the distribution of nociceptor subtypes [206,243].

Table 5 summarizes the commonly used electrophysiological devices for DPN assessment, which should be chosen based on the specific situation to ensure reliable diagnostic results. Future device development should focus on creating integrated devices with high sensitivity and multi-fiber measurement capacity, establishing individualized diagnostic criteria, and improving the patient experience. This approach will provide more comprehensive and efficient clinical solutions for early screening, diagnosis, and precise management of DPN (Fig. 6g).

**Table 5:** Electrophysiological devices for DPN assessment.

Electrophysiological assessment items	Main devices	Nerve types	Process	Measurement content	Advantages	Limitations	Ref
Nerve conduction research (NCS)	Electrical stimulator, recording electrodes, amplifier, electromyogram (EGM) devices, DPN check (no requirement for EGM)	Aβ fibers	Recording conduction velocity and amplitude after electrical stimulation of the nerve	CMAP, SNAP, DL, CV, F wave	The gold standard for DPN	Specialized equipment and procedures; high test cost; does not measure small diameter fibers; cannot distinguish between painful and painless DPN	[227,243,246]
Evoked potential (EP)	Heat generator, recording electrodes, amplifier, Electroencephalogram devices	A-δ and C fibers	Thermal stimulation triggers electrical neuronal responses and records the brain's response to the stimulation	Latency: Aδ fibers: 200-400 ms; C fibers: about 1000 ms	Able to assess the functional status of the CNS	Response to stimulation may be affected by many factors (eg, heating rate, stimulation, individual differences); cannot distinguish between peripheral and central pain pathway pathology; requires specialized equipment and personnel	[206,[231], [232], [233],^247^,^248^]
Electroencephalogram (EEG)	Multiple surface electrodes, amplifiers	Central nervous system (CNS)	Direct analysis of different frequency bands to reveal the functional characteristics of the brain in various states	Electrical activity in different frequency bands (δ、θ、α、β、γ)	High temporal resolution; able to analyze dynamic CNS functions	Results may be affected by multiple factors (e.g., attention shift); reliability as a pain biomarker remains to be verified	[234,[249], [250], [251]]
Electrochemical skin conductance (ESC)	Sudoscan: Surface electrodes, constant voltage source, measurement unit	C fibers	Measuring changes in skin conductance through sweat glands to reflect nerve function	Conductivity (in μS) is expressed as the ratio of the generated current to a constant DC stimulus	Short time, no need for professionals, non-invasive	Low specificity and uncontrolled differences between different populations and disease states; only measure the C fibers	[238,239,252]
Current perception threshold (CPT)	Neurometer®: Transcutaneous electrical stimulator, control unit, recording electrodes	Aβ, Aδ, C fibers	Determine the subject's threshold for electrical current to assess neurological function by gradually increasing the current intensity	Perception threshold current intensity (0.01 - 9.99 mA); the frequencies are 2000 (for Aβ), 250 (for Aδ), and 5 Hz (for C)	Ability to evaluate the function of 3 sensory nerves	Differences in populations lead to different sensitivity thresholds	[206,240,253]
Microneurography	A fine tungsten and reference electrode, amplifiers, and filters	C fibers	Neural activity is reflected by measuring the potential difference between the tungsten electrode and the reference electrode	Potential difference, latency (by different frequencies)	Simultaneous recording of multiple C-fibers and the identification of different C-fiber subtypes	Overly complicated operating procedures, not suitable for routine clinical diagnostics, require precise electrode placement and maintaining a high signal-to-noise ratio	[[243], [244], [245],^254^]

#### 5.1.3 Physiological assessment Devices

Physiological assessment devices for DPN include a range of non-invasive or minimally invasive devices. Blood glucose fluctuations are considered an independent risk factor for diabetic complications, and continuous glucose monitoring (CGM) provides a more precise assessment [255,256]. Compared with people with diabetes with painless DPN, those with painful DPN exhibited greater glucose variability, suggesting poorer diabetes control [257]. Furthermore, among patients with T2DM who had well-controlled HbA1c but no DPN, those who develop DPN show higher blood glucose variability [258]. HbA1c levels are associated with increased sensory latency in the upper limbs but not in the lower limbs [259]. Monitoring blood glucose levels only tracks the progression of DPN but does not provide a definitive diagnosis.

Skin biopsy is a safe, minimally invasive, and inexpensive diagnostic method considered the “gold standard” for diagnosing small fiber neuropathy. Morphometric analysis of cutaneous nerves by immunohistochemistry has the advantage of being reproducible and unaffected by neuropathy severity. Abnormal changes in cutaneous nerves were quantified by labeling with the pan-axonal marker protein gene product 9.5 (PGP9.5) to achieve early DPN assessment, but it was not possible to distinguish between painful and painless DPN [206,[260], [261], [262]]. To distinguish them, growth-associated protein 43 (GAP43) and PGP9.5 are often combined for analysis; a higher GAP43:PGP9.5 ratio was found in painful DPN than in painless DPN [261,263,264]. Additionally, painful DPN shows more positive Von Willebrand factor (vWF) expression than painless DPN [261,265]. Beyond structural indicators, recent research indicates that higher levels of peptidergic fibers (substance P and calcitonin gene-related peptide) in human skin and the presence of nociceptive Schwann cells (SOX10+/S100B+/AQP1+) are both associated with increased pain [[266], [267], [268]]. These markers show potential as biomarkers for future skin biopsies.

Sweat gland dysfunction is another characteristic of DPN, and the Neuropad is the only sweat gland function self-test device that can be used in primary care or at home [269,270]. The Neuropad is an adhesive pad impregnated with a blue cobalt chloride solution. When the cobalt encounters sweat, its color changes from blue to pink, and the degree of abnormality is determined by the color change (Fig. 6e) [271,272]. A colorimeter is used for a more accurate quantification [273]. Although the Neuropad has a sensitivity comparable to that of NCS in diagnosing DPN [274], it has a specificity lower than that of a 10-g monofilament [206]. Additionally, the temperature of the foot should be considered during the test, since warmer feet are more likely to show expected results [272].

Various blood biomarkers also serve as auxiliary tools in the assessment of DPN. Biomarkers of DPN can be divided into four categories: (1) AGE-related molecules, including methylglyoxal and glyoxalase I; biomarkers appear in the late stage of DPN, including (2) inflammation-related molecules (Toll-like receptors, miR-146a, adiponectin, etc.); (3) nerve damage-related molecules (nerve-specific enolase and signaling proteins); (4) neuroprotection-related molecules (nerve growth factor and heat shock protein 27) [275]. To detect these biomarkers, commonly used devices include PCR-based instruments and ELISA kits.

Table 6 summarizes the common physiological assessment devices. These devices offer the advantages of being non-invasive or minimally invasive and highly sensitive. However, they still have limitations in specificity and disease classification, such as distinguishing between painful and painless DPN. It is necessary to combine multimodal detection methods, including blood sugar, blood pressure, metabolic monitoring, neurological function testing, and blood molecular marker analysis, to develop a more accurate, stage-specific, and individually applicable assessment platform (Fig. 6g).

**Table 6:** Physiological devices for DPN assessment.

Physiological assessment methods	Main devices	Process	Measurement content	Measurement criteria	Advantages	Limitations	Ref
Blood glucose testing	Blood glucose meter	Blood collection or continuous monitoring of blood glucose levels	Changes in blood sugar levels and HbA1c content	Good control when HbA1c < 7%; blood sugar fluctuation range	Simple and continuous monitoring	Not for diagnosis, only for assessment of DPN progression	[[255], [256], [257],^259^,^276^]
Blood pressure testing	Blood pressure monitor	Continuous blood pressure monitoring	Diurnal blood pressure variation (ΔSBP, ΔDBP)	High probability of autonomic neuropathy when ΔSBP ≤0%, ΔDBP ≤5%	Simple and continuous monitoring	Not for diagnosis, only for assessment of DPN progression	[[277], [278], [279]]
Skin biopsy	Punch biopsy, immunostaining devices	Immunohistochemical staining (PGP9.5, GAP43) analysis after minimally invasive skin sampling	Statistics of epidermal nerve fiber density and GAP43/PGP9.5 ratio	Fewer IENFD indicates small fiber neuropathy; a high GAP43/PGP9.5 ratio suggests the possibility of pDPN	Minimally invasive and the gold standard for early detection of small fiber lesions	Unable to differentiate between painful and non-painful DPN; requires complex device support	[[260], [261], [262], [263], [264]]
Sweat gland function testing	Neuropad	Observe the color change after 10 min of patch application	Extent of color change	Pink is normal; partial pink or blue is abnormal	Simple (home-based), non-invasive, and high sensitivity	Low specificity, and correlated with foot temperature	[206,[269], [270], [271], [272], [273], [274]]
Blood biomarker testing	ELISA kits and PCR platform	Blood sampling or molecular testing	DPN-related molecules: AGE-related molecules, inflammatory, nerve injury factors (such as TLR4, IL-6, etc.)	According to the expression level of specific molecules	Analyze the progression of DPN at the molecular level, especially the potential pathology in the early stage	Wide variety of markers, no standardization, and individual differences	[275,[280], [281], [282]]

#### 5.1.4 Imaging assessment Devices

Imaging devices provide a non-invasive method to assess the detection and progression of DPN by visualizing structural and functional changes in peripheral nerves and related tissues. The cornea is the most densely innervated tissue in the body. Corneal confocal microscopy (CCM) quantified changes in corneal nerves to diagnose and stratify the severity of DPN, with high reproducibility and moderate to high sensitivity and specificity [205]. Alongside nerve cells, the maturation and density of corneal dendritic cells (DC) are linked to the progression of DPN. Patients with DPN exhibit higher densities of mature corneal DC [283,284]. The diagnostic performance of CCM in detecting people with diabetes with clinical DPN was comparable to that of intraepidermal nerve fiber density measurements in biopsy [285].

High-resolution ultrasound (HRU) dynamically scans and continuously observes the morphological changes of the entire nerve in real time, with unique diagnostic advantages in early DPN [286,287]. Ultrasound imaging reflects changes in nerve size, nerve echo texture, clarity of the perineurial margin, nerve bundle diameter, and vascularity. Patients with DPN have rich blood flow signals in the peripheral nerves and surrounding tissues [288]. Ultrasound detects abnormalities of peripheral nerves in people with diabetes with normal NCS results, but NCS is better than ultrasound in determining the severity of neuropathy [286].

Body temperature depends mainly on peripheral blood flow, and identifying asymmetric temperature distribution in the feet is an effective technique for early diagnosis of DPN [[289], [290], [291]]. Infrared thermal imaging technology (IRTI) is used to measure the distribution, state, and changes of the thermal field on the human body surface [289]. The nerves and blood vessels in the contralateral feet of DPN patients are damaged, resulting in increased temperature in the weight-bearing area, while normal people with diabetes show a symmetrical thermal pattern [290].

Hyperspectral imaging (HSI) is an objective, quantitative technique that allows for noninvasive assessment of the entire plantar skin within 5 min [289]. By using the short-wave infrared (SWIR) region (900–2000 nm), tissues exhibit characteristic absorption and reflectance characteristics (water, lipids, and collagen) [292]. Diabetes-induced protein glycation disrupts collagen structure, affecting skin, blood vessels, and nerves. Analysis of foot images after SWIR HIS acquisition determined the extent of DPN [292].

Magnetic resonance neurography (MRN) has well described the neurological changes in DPN, such as a smaller spinal cord cross-sectional area and altered vascular permeability (Fig. 6f) [293,294]. The T2 value of the tibial is an alternative noninvasive quantitative parameter for assessing DPN [295]. Proton MRI spectroscopy (1H-MRS) analysis also demonstrated changes in neuronal metabolites in patients with DPN, such as decreased creatine ratios and imbalances in glutamate/glutamine and γ-aminobutyric acid [293]. Additionally, diffusion tensor imaging (DTI) uses the anisotropy of water-molecule diffusion in tissues to display the morphology, structure, and shape of nerve fibers, and has been applied to the assessment of DPN [296].

Table 7 summarizes the common imaging assessment devices used for DPN. These devices non-invasively assess the structure, function, and metabolism of peripheral nerves and related tissues, but they mainly serve as a supplement to traditional tests rather than replacing conventional preclinical and electrophysiological tests. Although each method offers advantages in sensitivity and early detection of small fiber lesions, their application remains limited by the need for specialized procedures, the lack of standardized thresholds, and high costs. Future efforts should focus on establishing more unified diagnostic criteria (e.g., combining imaging and data analysis with machine learning) and identifying characteristic changes across different stages of DPN development (Fig. 6g).

**Table 7:** Imaging devices for DPN assessment.

Imaging methods	Main devices	Measurement content	Measurement criteria	Advantages	Limitations	Ref
Corneal confocal microscopy (CCM)	In vivo CCM with analysis software	Nerve fiber length/density/branching/tortuosity coefficient, rotation length of the corneal nerve, and maturity and density of corneal dendritic cells (DC)	ROC curves are drawn for each parameter tested to determine an optimal sensitivity/specificity CCM cutoff point for the DPN diagnostic; increased maturity and density of DC	Non-invasive, highly reproducible, and detects subclinical small fiber lesions	Image quality control; thresholds are affected by equipment or algorithms	[205,[283], [284], [285]]
High-resolution ultrasound (HRU)	High-frequency linear probe (12–18 MHz)	Nerve cross-sectional area (CSA), nerve echogenicity, fasciculation pattern, and intraneural vascularity	Enlarged CSA, increased hypoechoic area, altered fascicular pattern, increased vascularity	Visualized the entire nerve segment in real time; structural abnormalities and distribution can be found	Operator dependence; differences in standardized thresholds; less quantitative than NCS for severity	[[286], [287], [288]]
Infrared thermal imaging (IRTI)	Infrared thermal imager	Foot surface temperature distribution and asymmetry	A common threshold is a contralateral temperature difference of >2.2 °C	Non-invasive, fast, and can be used for remote/home monitoring and risk warning	Highly affected by environmental and individual differences, its use in DPN grading remains to be verified	[[289], [290], [291]]
Hyperspectral Imaging (HIS)	Hyperspectral scanner with analysis software	water, lipid, collagen content, and oxygenation patterns	Take the average value of the hyperspectral waveform of all pixels in the plantar skin for judgment	Objective, sensitive, rapid, non-invasive, and remote, capable of identifying small fibers	High equipment and algorithm threshold, and uncertainty in thresholds and models	[289,292]
Magnetic resonance neuroimaging (MRN, T2-mapping, DTI, 1H-MRS)	MRI scanner with neurography sequences, diffusion tensor imaging, and MR spectroscopy	Neural T2 values, diffusion indices (FA and ADC values), and nerve-related metabolites	T2 values increase; FA decreases, while ADC increases; neurometabolic balance is disrupted	Non-invasive, quantitative, microstructural assessment, metabolic assessment	High cost and time-consuming; challenges in small nerve resolution/motion artifacts	[[293], [294], [295], [296]]

### 5.2 Emerging technologies for DPN monitoring

Emerging DPN monitoring technologies are rapidly developing to overcome the limitations of current commercial devices, which primarily rely on discrete, clinically based measurements. Recent advances in artificial intelligence (AI), multimodal data integration with wearable devices, biosensors, and telemedicine platforms are enabling continuous, multi-parameter, and personalized monitoring of DPN. Two key areas of focus are: 1) AI-driven approaches that can reveal complex, nonlinear relationships between clinical variables that traditional statistical methods may overlook, leveraging data from large-scale clinical test results for predictive and personalized monitoring, enabling risk modeling; 2) wearable devices that allow continuous, wireless, highly specific, and non-invasive/mini-invasive monitoring of DPN-related physiological parameters during daily life.

#### 5.2.1 AI-Driven approaches for DPN monitoring

AI is transforming the detection and management of DPN by enabling automated, data-driven approaches that surpass traditional monitoring methods. Through machine learning (ML) and deep learning (DL), AI systems can monitor DPN progression by analyzing complex medical images, physiological results, and biological signals to identify subtle signs of nerve damage that might escape human observation. These applications support accurate and reproducible assessments, offering new opportunities for early diagnosis, risk prediction, and personalized treatment of DNP.

The integration of AI into imaging techniques has become a powerful method in monitoring DNP. Automated measurement of corneal nerve fiber density and length using convolutional neural networks (CNNs) achieves diagnostic accuracy similar to that of expert graders while offering reproducibility suitable for long-term follow-up [297]. DL models (such as CNN and ResNet-50 with Grad-CAM) outperform traditional software in quantifying corneal nerve fiber density, length, and branching [298]. A fully automated DL segmentation algorithm was combined with an adaptive neuro-fuzzy inference system (ANFIS) to classify patients with and without DPN based on CCM images [299,300]. Preston et al. successfully confirmed the feasibility of using an AI-based DL algorithm (DLA) for diagnosis, without requiring image segmentation before classification or a large dataset to train the model [298].

In addition to imaging, AI and ML have shown value in analyzing clinical physiological test results for DPN. After analyzing DPN patient data with a logistic regression model using ML, it was found that the Extreme Gradient Boosting (XGBoost) algorithm had the highest diagnostic performance (Area under the curve, AUC = 0.69), with the neutrophil-to-lymphocyte ratio, diabetes duration, glycated hemoglobin (HbA1C), and renal function being the strongest predictors [301]. The AI model also predicted DPN based on vibration perception threshold and other clinical variables, such as total cholesterol and creatinine ratio, for risk assessment of DPN [302]. Meanwhile, a soft computing decision support system could make decisions for monitoring DPN severity in patients with T2DM using real-time clinical and demographic data [303].

The system for perceiving and analyzing biological signals (biomechanical, electrophysiological, and electrochemical) offers another promising area for early detection of diabetic neuropathy using AI strategies. ML classifiers such as k-nearest neighbor (KNN), support vector machine (SVM), and random forest (RF) have been used to analyze gait data from EMG and ground reaction forces, achieving a classification accuracy of 98.7%, which can precisely identify DPN [304]. DL combined with RF was employed to analyze TCSS and NCS data from 394 participants, resulting in an AUC of 0.98 for diagnosing DPN and 0.93 for predicting DPN within a year [305]. Additionally, ML successfully identified key risk factors for DPN, including foot and hand ESC, HbA1C, and duration of diabetes, based on Sudoscan ESC data [306].

Table 8 summarizes current AI-driven methods for DPN monitoring. AI has demonstrated significant promise and performance in DPN applications, but some areas need improvement. Although decision trees, RF, and neural networks are effective tools, research on T1DM remains limited [307]. Moreover, most studies rely on small or single-center datasets that lack demographic and ethnic diversity. Future models should be developed and validated using large, multi-center, demographically diverse datasets and cross-institutional training to improve generalization. Key challenges still exist in data standardization, interpretability, ethical oversight, and model transferability across different populations [308]. Furthermore, the development of multimodal AI models in DPN research remains limited, and integrating data, such as images, clinical records, and biosignals, is necessary for more comprehensive monitoring. Integrating AI with wearable devices to enable personalized, continuous monitoring also represents a promising direction [308]. Finally, a notable gap exists in AI applications that combine gene data, receptor sequences, cell multi-omics, and related information [[309], [310], [311], [312], [313]]. Addressing these challenges through interdisciplinary collaboration and large-scale clinical validation is essential to realize the full clinical potential of AI-driven DPN monitoring.

**Table 8:** AI-driven approaches for DNP monitoring.

AI Application Domain	Main Methods/Algorithms	Input Data	Target Outcome	Performance	Advantages	Limitations	Ref
Imaging (CCM)	Liverpool Convolutional Neural Network (LCNN), Liverpool Deep Learning Algorithm (LDLA); ResNet-50, MobileNet, MobileNetV2; DL with neuro fuzzy inference system (ANFIS)	CCM images of corneal nerve fibers	Automated quantification and classification of diabetic small-fiber neuropathy	Area under curve (AUC) = 0.83, Sensitivity 0.68; Specificity 0.87; PN + recall = 0.83, precision = 1.0, F1 = 0.91; AUC up to 0.95	Non-invasive, objective, and highly reproducible; potential replacement for skin biopsy	Requires high-quality images and standardized CCM protocols; small sample sizes limit generalization	[[297], [298], [299], [300]]
Clinical physiological test results	Support Vector Machine (SVM) and XGBoost; Residual Multi-Layer Per-ceptron (ResMLP), Long Short-term Memory Networks (LSTM), Deep Neural Networks (DNN), and One-dimensional Convolution Neural Networks (1D-CNN); Artificial Neural Network (ANN)	Clinical and laboratory variables (HbA1C, duration, renal function)	Early prediction and screening for DPN; risk and severity level of DPN	XGBoost AUC = 0.69; SVM AUC = 0.62; accuracy up to 99%; accuracy >70%	Uses routine clinical data; interpretable; scalable to Electronic Health Record (EHR) integration	Difference in accuracy; limited by data quality and population bias	[[301], [302], [303]]
Gait and electrophysiological monitoring	Eight ML (machine learning) models: Discriminant analysis classifier (DAC), Ensemble classification model (ECM), Kernel classification model (KCM), k-nearest neighbor model (KNN), Linear classification model (LCM), Naive Bayes classifier (NBC), SVM, Binary decision classification (BDC); Random forest (RF) combined with ML	EMG & ground reaction force (GRF) signals; NCS; TCNS	Detection and longitudinal prediction of DPN	Accuracy up to 98.68%; AUC up to 0.98	Integrates biomechanical and electrophysiological signals; personalized and continuous monitoring; suitable for wearable or remote health applications	Require specialized hardware; high cost; motion variability affects accuracy	[304,305]
Autonomic function	Logistic Regression, RF, Lazzy classifiers	Sudoscan ESC, metabolic, and clinical features	Diagnosis and prediction of DAN	Accuracy up to 97%	Simple, rapid, and low-cost tool; supports personalized risk management	Device-dependent calibration; limited cohort diversity; less validated for long-term monitoring	[306]

#### 5.2.2 Wearable Devices for DPN monitoring

With advances in wearable and flexible electronics, wearable devices are emerging as promising technologies for noninvasive DPN monitoring. Current research on wearable devices for DPN primarily focuses on gait and load monitoring using inertial and plantar pressure sensors. Since DPN is associated with both electrophysiological abnormalities and sweat gland dysfunction, wearable technologies capable of electrophysiological signal acquisition and sweat-based biochemical sensing will also be crucial for future DPN monitoring applications.

Wearable inertial sensors can diagnose DPN by recording and analyzing gait parameters [314]. A DPN diagnostic framework using gravity-sensitive inertial measurement units (IMUs) and angular velocity-measuring gyroscopes exhibited differences in serial tasks performance and walking speed [314]. However, the results are susceptible to numerical integration errors, noise, drift, bias, temperature changes, and magnetic interference [315,316]. Therefore, filter selection is crucial, yet few studies have reported or examined the impact of filter settings on gait event detection [316,317]. Moreover, comfort and the effects of sweat and temperature on sensors should also be considered during long-term monitoring [318].

**Fig. 7:**
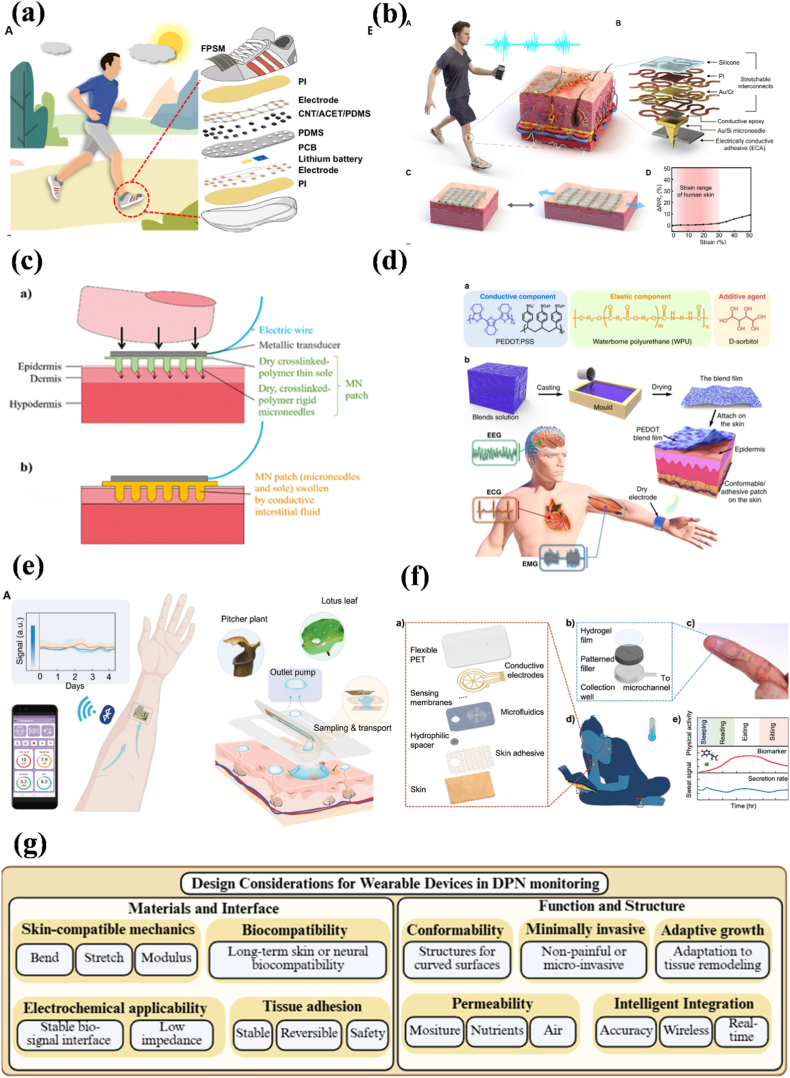
a) The self-powered smart insole [320]; b) The stretchable microneedle adhesive patch (SNAP) providing excellent skin penetrability and a robust electromechanical skin interface for prolonged and reliable EP monitoring under varying skin conditions [325]; c) The crosslinked-hydrogel MN-based electrodes for biopotential measurements [329]; d) The adhesive electrode on the skin for epidermal biopotential detections such as electrocardiography (ECG), electromyography (EMG), and electroencephalography (EEG) [330]; e) The bioinspired microfluidic sweat sensor system for extended sweat sampling, transport, and multiday sweat analysis [337]; f) The microfluidic sweat analysis patch for continuously monitoring both sweat secretion rate and composition for long-term without external sweat stimulation [338]; g) design considerations for wearable devices in DPN monitoring, created by Biorender.

DPN patients consistently exert more pressure on their feet while walking compared to healthy individuals. Therefore, wearable pressure sensor devices, such as engineered insoles, can be used to collect plantar pressure data and monitor DPN patients [319]. The plantar pressure in the forefoot area of DPN patients is significantly increased [314]. Current pressure sensors still face several challenges. For example, resistive sensors exhibit a nonlinear response to pressure, making data analysis complex; capacitive sensors have weak anti-interference capabilities, complex measurement circuits, and low sampling rate and accuracy; and piezoelectric and triboelectric sensors are limited to measuring only dynamic pressure signals [320]. Additionally, the power supply remains an essential considerations [321]. An integrated self-powered wireless smart insole system developed by Wang et al. addressed the issue of nonlinear response, low stability, and energy limitations, representing a potential next generation of wearable pressure sensor devices (Fig. 7a) [320]. Because of the fluctuation of foot temperature among DPN patients, temperature sensors have been combined with pressure sensors to create engineered insoles [314,322]. Smart socks have also been used for remote patient monitoring [323]. Skin electronic sensors that can accurately distinguish between temperature and pressure stimuli are crucial. Yu et al. developed a flexible temperature-pressure dual sensor based on a three-dimensional spiral thermoelectric Bi2Te3 thin film, which exhibited excellent pressure and temperature sensing performance [324].

Wearable electrophysiological devices, such as NCS, ECG, EMG, and EEG, are widely used in clinical DPN assessments. Nonetheless, traditional devices still require improvements. For instance, microneedle electrodes often have poor skin contact. Kim et al. developed a stretchable microneedle patch for long-term signal monitoring that was highly reliable, unaffected by skin conditions, and easy to use without preparation, offering a higher signal-to-noise ratio than flexible microneedle and gel electrodes (Fig. 7b) [325]. While electrodes in NCS and EMG tests mainly focus on signal acquisition, nerve stimulation remains essential. Zhang et al. introduced hydrogel electrodes optimized with the Hofmeister effect, featuring low modulus, high stretchability, and high viscosity. These properties improved signal continuity, reduced distortion, higher signal-to-noise ratio, and lower stimulation voltage, enhancing both safety and user comfort during nerve conduction tests [326]. Traditional ECG devices often compromise comfort. Composite skin electrodes crafted from materials such as silver nanowire/TPU scaffolds, PEDOT:PSS, and CNT hybrid textiles deliver both comfort and excellent conductivity [327,328]. In long-term EEG and ECG monitoring, wet gel electrodes can leak or dry out, leading to unstable signals [329]. Juillard et al. developed a dry hydrogel microneedle electrode that penetrates the skin to contact the dermal interstitial fluid, providing more stable measurements than wet gel electrodes (Fig. 7c) [329]. Furthermore, dry electrodes are essential for sustained biopotential recording. Zhang et al. created a durable dry electrode capable of reliable epidermal biopotential monitoring during extended exercise and of capturing high-quality ECG, EMG, and EEG signals on both wet and dry skin under various conditions, including physical activity (Fig. 7d) [330].

Metabolites in sweat offer a valuable source of information for non-invasive skin biosensor monitoring [331]. Cheng et al. developed a wearable electroenzymatic sensor that replaced traditional Prussian blue and measured multiple biomarkers, such as glucose, lactate, and choline, with high sensitivity, selectivity, stability, and operational reliability in sweat samples [332]. A hybrid epidermal biosensor (HEB) patch that concurrently measured sweat glucose and electrocardiogram was also introduced [333]. Antibodies play a crucial role in detecting hormones in sweat by modifying conductive wire electrodes with L-cysteine, gold nanoparticles, and MXene before antibody immobilization to increase surface area and electron transfer capacity, resulting in a wide linear range and low detection limits [331,334]. A conductivity sensor for IL-6 made of oxygen-deficient zinc oxide (ZnO) showed high selectivity, detecting IL-6 concentrations 100 times lower than healthy levels [335]. Zhao et al. developed a skin-like, drift-free biosensor based on a stretchable diode-connected organic field-effect transistor that reduced signal distortion by up to two orders of magnitude, making it suitable for aptamer-based cortisol sensing, enzyme-based glucose detection, and ion-selective membrane-based sodium ion potential sensing [336]. The wearable microfluidic-based sweat sensor performed real-time molecular analysis for several days, measuring uric acid, xanthine, and alcohol (Fig. 7e) [337]. Microfluidic technology was also employed to reduce evaporation. When combined with a hydrophilic filler that rapidly absorbs sweat, this enabled continuous sweat monitoring at rest (nL min^−1^ cm^−2^) (Fig. 7f) [338].

Although inertial and pressure-based wearable sensors for DPN have been developed, variability between devices remains a key challenge in achieving consistent measurement. For example, systems with eight pressure sensors strategically placed on the sole offer a practical approach, whereas systems with up to 960 sensors are also available, highlighting the need for consistent measurement standards across people with diabetes [339]. Future efforts in cross-sensor calibration and accuracy validation for DPN will face additional complexity due to variations in skin thickness, body fat percentage, and user movement, all of which can influence sensor performance [331]. In DPN, factors such as compromised skin integrity, low foot sweating, and peripheral nerve degeneration create unique mechanical, biochemical, and electrochemical challenges for wearable sensor systems [340]. Furthermore, neuropathy often begins in extremities, which experience significant curvature and mechanical stress, placing high demands on the materials used in sensor systems. Therefore, future wearable devices for DPN monitoring should ensure stable adhesion, a low-impedance interface, fatigue resistance, and moisture protection, while also improving measurement accuracy and supporting real-time, continuous Bluetooth data transmission. The device design should also prioritize comfort, breathability, and precise sensing on irregular surfaces (Fig. 7g). Finally, although wearable bioelectronics and ML approaches can quantify the impact of DPN, future research should focus on integrating therapeutic and functional restoration capabilities [341]. For example, the sensory nerve prosthesis, NeuroStep, has been shown to enhance gait and decrease neuropathic pain by electrically stimulating the foot through specially designed insoles [342]. Additionally, wearable devices are now making sensory replacements more feasible. Flavin et al. developed a programmable, skin-compatible, bistable transducer that functions as a sensory replacement device, potentially improving the lives of those with visual and proprioception impairments [343].

## 6 Conclusion

The pathophysiology of DPN is complex, posing a significant clinical challenge. Current treatments primarily focus on pain relief rather than disease reversal, underscoring the urgent need for approaches that bridge mechanistic understanding with innovative therapeutic strategies.

Recent clinical trials investigating pharmacological agents and biological therapies have shown promise in pain relief, neuroprotection, anti-inflammatory effects, and tissue regeneration. Animal models for preclinical studies are vital for understanding DPN mechanisms and the effects of treatment. To improve translational relevance, these models must be carefully selected and refined to accurately replicate the clinical features of DPN at various stages and symptoms. Future directions might involve enhancing stage- and phenotype-specific DPN animal models to better reflect clinical features, and incorporating metabolic, vascular, and aging factors to improve preclinical studies' ability to capture disease heterogeneity and forecast clinical outcomes. Advances in neurobiology and regenerative medicine have deepened our understanding of DPN, while biomaterial-based drug delivery systems and microfluidic platforms are advancing therapeutic development, disease modeling, and mechanistic research. Although biomaterial-based drug delivery systems have enhanced targeting, sustained release, reduced systemic toxicity, and enabled combination therapies, several challenges remain. These include limited clinical translation, lack of stage-specific targeting, incomplete systemic metabolic coordination, insufficient attention to nerve-wound interactions, and restricted diversity in animal models. Future research may focus on integrating biomaterial-based drug delivery systems with animal models that exhibit specific pathological mechanisms and phenotypic traits, enabling targeted treatment at various stages of DPN. Developing delivery systems with controllable and targeting capabilities that can provide long-lasting, stable therapeutic effects during the early and middle stages of DPN could be a promising approach to DPN therapy. Likewise, microfluidic platforms provide precise control over the neural microenvironment *in vitro*, enabling personalized, detailed studies of disease mechanisms and therapeutic responses. However, they face limitations, such as inadequate target organ modeling, incomplete replication of disease microenvironments, and the absence of comprehensive drug screening platforms. Future DPN microfluidic platforms can be further developed by integrating multi-organ interfaces (DPN-on-a-chip), immune and vascular modules, and standardized drug screening processes, thereby more accurately simulating the disease microenvironment and therapeutic effects. While therapeutic development is crucial, the creation of bioengineered devices for DPN assessment and monitoring is equally essential for effective disease management. These devices allow quantification of neuropathy severity, enable early detection, and assess treatment success. Incorporating AI into such systems enhances precision and provides critical feedback, linking clinical observations of DPN progression with decision-making. Nonetheless, current assessment devices lack standardized diagnostic criteria, and wearable monitoring devices must better account for factors associated with DPN, such as impaired skin integrity, reduced sweating, and nerve degeneration. Future work should consider establishing standardized, DPN-stage-specific assessment and diagnostic metrics, and integrating multimodal diagnostic data along with continuous wearable monitoring into a single AI platform to support long-term monitoring of DPN progression and to enable personalized, adaptive disease management across different clinical stages. Carefully designed studies to address these gaps will accelerate the development of effective therapies for DPN.

Beyond incremental advancements in various fields, the future management of DPN will increasingly depend on the synergistic integration of biomaterial-based drug delivery systems, microfluidic platforms, AI, and assessment and monitoring devices. Microfluidic platforms can be used efficiently to evaluate the therapeutic effects, release kinetics, and target organ interactions of biomaterial systems, enabling iterative *in vitro* optimization before *in vivo* application. At the same time, AI-driven big data analysis and functional evaluation of biomaterials and drugs will assist in screening specific drug carriers and delivery materials. The integration of biomaterial-based delivery systems with monitoring devices further enables the development of closed-loop systems, in which on-demand drug release can be triggered by real-time electrophysiological or biochemical signals, thereby enabling dynamic regulation of DPN progression. Furthermore, combining microfluidic technology with evaluation and monitoring platforms enables high-precision analysis of patient biofluids and biomarkers, thereby supporting standardized workflows. Effectively integrating these technologies is a crucial area of research for the future management of DPN.

Integrating mechanism-driven therapies, improved animal models, advanced biomaterial-based drug delivery systems, microfluidic platforms, and intelligent assessment tools will establish a comprehensive, closed-loop framework for DPN treatment and management. Collaboration among clinicians, bioengineers, materials scientists, and pharmaceutical researchers is crucial to overcoming existing challenges, transitioning from symptom control to disease reversal, and ultimately improving the quality of life of people with diabetes.

## CRediT authorship contribution statement

**Zhi He:** Writing – original draft, Investigation, Conceptualization. **Jie Diao:** Writing – original draft. **Frederick G. Hamel:** Writing – review & editing, Supervision, Conceptualization. **Bin Duan:** Writing – review & editing, Supervision, Project administration, Funding acquisition, Conceptualization.

## Ethics approval and consent to participate

There are no human or animal subjects in this review.

## Declaration of AI-assisted technologies in the writing process

During the preparation of this work, the authors used ChatGPT in order to polish the content. After using this tool, the authors reviewed and edited the content as needed and take full responsibility for the content of the published article.

## Declaration of competing interest

The authors declare no conflict of interest.
